# The Systemic Imprint of Growth and Its Uses in Ecological (Meta)Genomics

**DOI:** 10.1371/journal.pgen.1000808

**Published:** 2010-01-15

**Authors:** Sara Vieira-Silva, Eduardo P. C. Rocha

**Affiliations:** 1 Microbial Evolutionary Genomics, Institut Pasteur, CNRS, URA2171, Paris, France; 2 Atelier de BioInformatique, UPMC Univ Paris 06, Paris, France; University of Arizona, United States of America

## Abstract

Microbial minimal generation times range from a few minutes to several weeks. They are evolutionarily determined by variables such as environment stability, nutrient availability, and community diversity. Selection for fast growth adaptively imprints genomes, resulting in gene amplification, adapted chromosomal organization, and biased codon usage. We found that these growth-related traits in 214 species of bacteria and archaea are highly correlated, suggesting they all result from growth optimization. While modeling their association with maximal growth rates in view of synthetic biology applications, we observed that codon usage biases are better correlates of growth rates than any other trait, including rRNA copy number. Systematic deviations to our model reveal two distinct evolutionary processes. First, genome organization shows more evolutionary inertia than growth rates. This results in over-representation of growth-related traits in fast degrading genomes. Second, selection for these traits depends on optimal growth temperature: for similar generation times purifying selection is stronger in psychrophiles, intermediate in mesophiles, and lower in thermophiles. Using this information, we created a predictor of maximal growth rate adapted to small genome fragments. We applied it to three metagenomic environmental samples to show that a transiently rich environment, as the human gut, selects for fast-growers, that a toxic environment, as the acid mine biofilm, selects for low growth rates, whereas a diverse environment, like the soil, shows all ranges of growth rates. We also demonstrate that microbial colonizers of babies gut grow faster than stabilized human adults gut communities. In conclusion, we show that one can predict maximal growth rates from sequence data alone, and we propose that such information can be used to facilitate the manipulation of generation times. Our predictor allows inferring growth rates in the vast majority of uncultivable prokaryotes and paves the way to the understanding of community dynamics from metagenomic data.

## Author Summary

Microbial minimal generation times vary from a few minutes to several weeks. The reasons for this disparity have been thought to lie on different life-history strategies: fast-growing microbes grow extremely fast in rich media, but are less capable of dealing with stress and/or poor nutrient conditions. Prokaryotes have evolved a set of genomic traits to grow fast, including biased codon usage and transient or permanent gene multiplication for dosage effects. Here, we studied the relative role of these traits and show they can be used to predict minimal generation times from the genomic data of the vast majority of microbes that cannot be cultivated. We show that this inference can also be made with incomplete genomes and thus be applied to metagenomic data to test hypotheses about the biomass productivity of biotopes and the evolution of microbiota in the human gut after birth. Our results also allow a better understanding of the co-evolution between growth rates and genomic traits and how they can be manipulated in synthetic biology. Growth rates have been a key variable in microbial physiology studies in the last century, and we show how intimately they are linked with genome organization and prokaryotic ecology.

## Introduction

Maximal growth rates are central to microbial life-history strategies [1]–[9]. Among host-associated bacteria, competition often results in increased virulence through selection for higher growth rates as these have an outstanding role in the trade-off between rapid horizontal dissemination and slow clearance from the host [10],[11]. Highly infectious bacteria are associated with high maximal growth rates, e.g. enterobacteria, whereas bacteria producing chronic infections, e.g. mycobacteria, typically grow slowly under optimal conditions. The rapidity of spread of some bacteria poses a problem of urgency in antibiotic treatment, rendered more difficult by arising multiple resistances [12]. But slow growing bacteria sometimes also pose a therapeutic problem, as many antibiotics are ineffective in very slow growing cells [13]. Among free-living bacteria there is also a trade-off between fast growth in copiotrophs and scavenging potential in slow-growing oligotrophs [4],[14],[15]. Copiotrophic bacteria tend to have low affinity transporters and abundant gene expression machinery allowing fast growth in periods of feast, while enduring starvation in periods of famine where much of the protein synthesizing machinery is degraded [16]. Slow growing oligotrophs have high affinity transporters allowing them to thrive even under very small nutrient concentrations, but these become saturated or even toxic at high nutrient concentrations leading to their selective exclusion by fast growers in rich environments [17]. Because growth rates are outcomes and constraints of microbial life-history strategies, it is important to understand the mechanisms allowing fast growth and how they are imprinted by natural selection in genomes. Inversely, it would be extremely useful to predict maximal growth rates from sequence alone. This would allow establishing generation time predictions for the vast numbers of unknown or uncultivated bacteria for which we lack such information.

Classical studies in *E. coli* physiology have uncovered the physiological changes concomitant with fast growth (reviewed in [18]). When *E. coli*'s generation time decreases from 100 to 24 min, cellular RNA polymerases (RNAP) are multiplied by 15 and ribosomes by 10. A large fraction of the additional transcription capacity is used to produce stable RNA (rRNA and tRNA). While the rate of synthesis also increases, it does so at much more moderate rates, e.g. elongation is faster by 40% for RNAP and 75% for ribosomes, which then attain maximal translation capacity. Thus, high growth rates result more from the increase in the production of the gene expression machinery than from its increasing productivity. At high growth rates, about 74% of all *E. coli* transcription concerns the production of stable RNA. To allow for such high levels of expression stable RNA genes tend to be in multiple copies in fast growing bacteria [19]. This multiplicity of rRNA operons constitutes a metabolic burden at lower growth rates [20].

In fast growing *E. coli* B/r, a replication round starts every 20 minutes, corresponding to the cell's minimal doubling time. Yet, replication of the chromosome takes ∼45 minutes [21]. This is possible because multiple rounds of replication can occur concurrently. The start of a new replication round before the previous one has finished doubles the number of regions around the replication origin in the cell. In cells with three simultaneous rounds of replication, genes the near the origin are thus 8 times more abundant in the cell than the genes near the terminus of replication. In the absence of negative feedback regulatory control, replication associated gene-dosage effects result in higher gene expression levels near the origin of replication [22]–[24]. Since genes coding for the translation and transcription machineries are under particularly strong demand at times of fast growth, there is a strong selection for their positioning near the origin of replication in fast growing, but much less so in slow growing, bacteria [25].

Even if tRNA concentration in the cell increases with growth rates, the tRNA/ribosome ratio decreases by 50% when comparing slow and fast growing *E. coli* [26]. The tRNA pool becomes limiting at very high growth rates. Thus, its quick turnover at ribosomes is under strong selection. This can be optimized if codons of highly expressed genes under fast growth recruit the most abundant tRNA in the cell [27]. Such codon usage bias, i.e. differential preference of some synonymous codons over others, is therefore as strong as the gene is highly expressed [28],[29]. It is also stronger for fast growing bacteria because of the above-mentioned decrease of tRNA/ribosome at higher growth rates and because in these conditions the few percent most highly expressed genes account for a larger fraction of all gene expression. Codon usage bias is thus thought to result from selection for accurate and fast translation by maximizing the recruitment of the most abundant tRNAs into ribosomes [30]. The highly significant role of translation and its machinery in the cell budget of fast growing bacteria makes codon usage bias a good predictor of gene expression levels under exponential growth [31],[32].

There have been studies on the association between maximal growth rates and rRNA operon [1],[19],[33],[34] and tRNA [35],[36] multiplicity, replication-associated gene dosage [25],[37] and codon usage biases [35],[38]. All these factors are thought to imprint genomes in accordance with the microbe's maximal growth rates. Previous studies focused on only one of the traits in one or few genomes and sometimes using coarsely binned growth data. To understand the relative role and importance of each factor and be able to manipulate growth rates more integrative studies are required. Unfortunately, the paucity of physiological data for the vast majority of microbes precludes the use of mechanistic models that can only be parameterized in *E. coli* [39]. Hence, we decided to use an empirical approach to answer the following questions: What is the association of each growth-related trait with maximal growth rates? How inter-correlated are they? What is their predictive power? Can we use the growth-related genomic traits to test ecological hypothesis with metagenomic data?

## Results/Discussion

### Genomic signatures of adaptation to fast growth

Following a previous work [35], we extracted from primary literature 214 minimal generation times (d) of species of bacteria and archaea (Table S1). We used this data to assess how genomic traits correlate with minimal generation times. We started by analyzing its correlation to genome size. Historically, microbial genomes have been viewed as short and compact due to selection for rapid replication and fast growth. In agreement with previous work [40],[41], we found no evidence for a positive correlation between minimal generation time and genome size or genome density (Spearman correlations ρ = −0.10 and −0.08, p-value = 0.13 and 0.24). The reasoning that smaller genomes allow for quicker replication is belied by the observation that replication can be initiated before the previous rounds have finished. There is thus no necessity for a direct correlation between genome size and minimal generation time, as observed.

**Figure 1:**
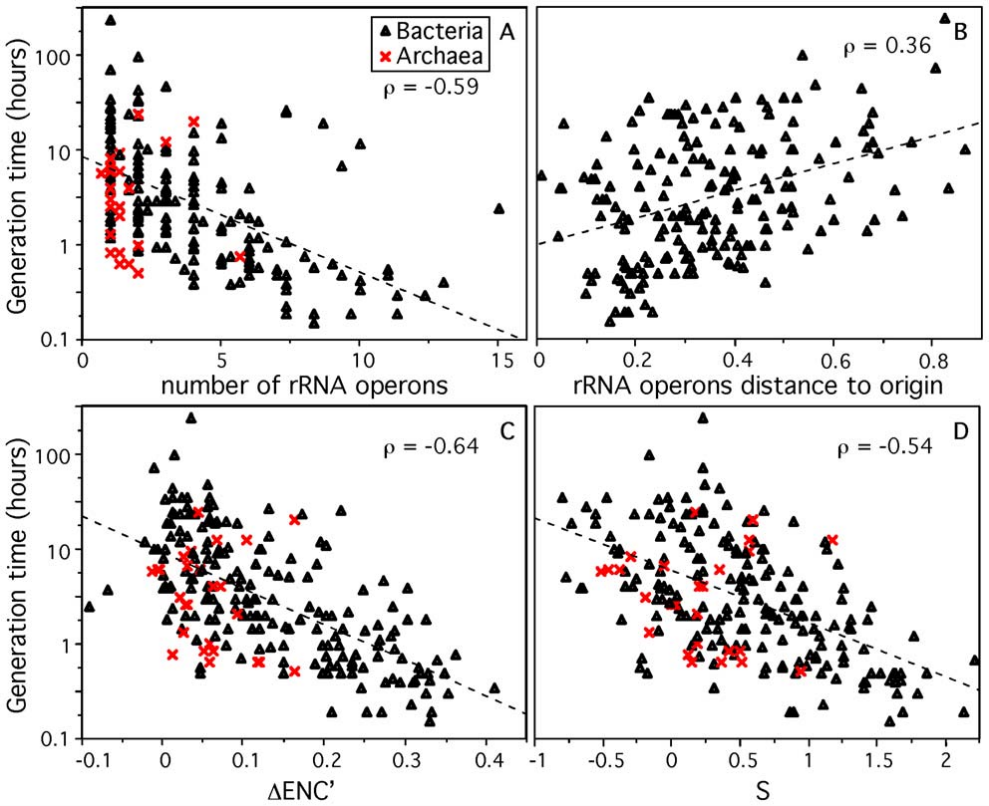
Genomic signatures correlated to minimum generation time (d) for 214 prokaryotes. Correlation between d and (A) the number of rRNA operons in the genome, (B) the relative distance from the origin of replication to rRNA genes (excluding species with no retrievable origin), 0.5 corresponds to half the replicon, (C,D) codon usage bias indices ΔENC′ [35] and S [46]. Spearman correlations are given (ρ) with all p-values<0.0001. Dashed lines represent the trend of the correlation.

As expected, we found an increase in copy number of rRNA (Figure 1) and tRNA genes (Figure S1) with decreasing minimal generation times (ρ = −0.59 and ρ = −0.51, all p-value<0.0001). The multiplicity of the subset of nearly ubiquitous tRNAs (ubi-tRNA, listed in Table S2), which in most species match the most favored codons [35], is more correlated with d than the other tRNA genes (ubi-tRNAs and non-ubi-tRNAs respectively, ρ = −0.54 and ρ = 0.13, p-value<0.0001 and p-value = 0.06, Figure S1). While many enterobacteria contain two copies of the highly expressed elongation factor Tu [42], we found no systematic trend for duplication of highly expressed protein coding genes in fast growers. Since each mRNA is translated ∼100 times [18], multiple copies of ribosomal protein coding genes would only be required to match the expression of rRNAs if the latter was present in excess of 100 copies. However, in our dataset, and in the rRNA Operon Copy Number Database [43], the maximal number of rRNA operon copies is 15 for *Photobacterium profundum*.

As described above, gene dosage of highly expressed genes can be increased transiently when these genes are located near the origin of replication in fast growing cells. Indeed, a positive correlation was found between minimum generation time and the relative distance to the origin of replication of rRNA genes (ρ = 0.36, Figure 1), RNA polymerase genes (ρ = 0.42), ribosomal proteins coding genes (ρ = 0.42), tRNA (ρ = 0.35) and ubi-tRNA (ρ = 0.41) genes (Figure S2) (all p-values<0.0001). Hence, our data supports previous work suggesting that high growth rates are correlated with high transient or stable gene dosage in highly expressed genes associated with translation and transcription [25]. The importance of gene multiplicity, based on gene deletion studies, has been attributed to selection for quick start of exponential growth, not for its maintenance [1],[2],[19],[44]. These two effects are tangled in genome organization because selection for fast growth is usually associated with selection for quick start of exponential growth in copiotrophic bacteria enduring feast and famine regimes [1],[16],[45]. Once replication has started, the replication-associated gene dosage effect ensures that rRNAs are in much higher copy number in the cell than expected given their gene multiplicity. This makes the 7 copies of rRNA genes in *E. coli* to effectively increase in the cell by a factor of 5 under maximal growth [18]. Thus, gene multiplicity and replication-associated gene dosage can be seen as complementary, with the former being essential for the start of exponential growth and affecting stable RNA genes, and the latter ensuring high cellular concentration of translation and transcription-associated highly expressed genes under stable growth, thus affecting both RNA and protein coding genes.

Finally, two previously proposed indices of codon usage bias in highly expressed genes ΔENC′ [35] and S [46] correlate negatively with d (respectively, ρ = −0.64 and ρ = −0.54, p-value<0.0001, Figure 1). For the calculation of these indices we used the ribosomal proteins as the set of highly expressed genes under exponential growth (see Materials and Methods), as this is frequently done [29],[32],[35]. The ubiquity and high conservation of ribosomal proteins facilitate the identification of this set of genes in the subsequent metagenomic analyses. We tested that the results remained qualitatively similar when using other highly expressed genes under exponential growth, such as elongation factors or RNA polymerase genes (data not shown). Although ΔENC′ corrects for the influence of the G+C content of the genome on codon usage bias, we verified that G+C content is not correlated with minimal generation time (ρ = 0.06, p-value = 0.39) nor with ΔENC′ (ρ = 0.09, p-value = 0.24). Incidentally, genomic G+C content correlates with genome size (ρ = 0.61, p-value<0.0001) [47],[48]. The correlation between codon usage bias and minimum generation time is attributable to the selective pressure acting on highly expressed genes for the use of translationally optimal codons in these genomes where few genes correspond to the vast majority of gene expression. While experimental work has shown the advantages of optimizing codon usage bias for expression of heterologous proteins [49], our results suggest that optimization of highly expressed genes should lead to higher growth rates.

**Figure 2:**
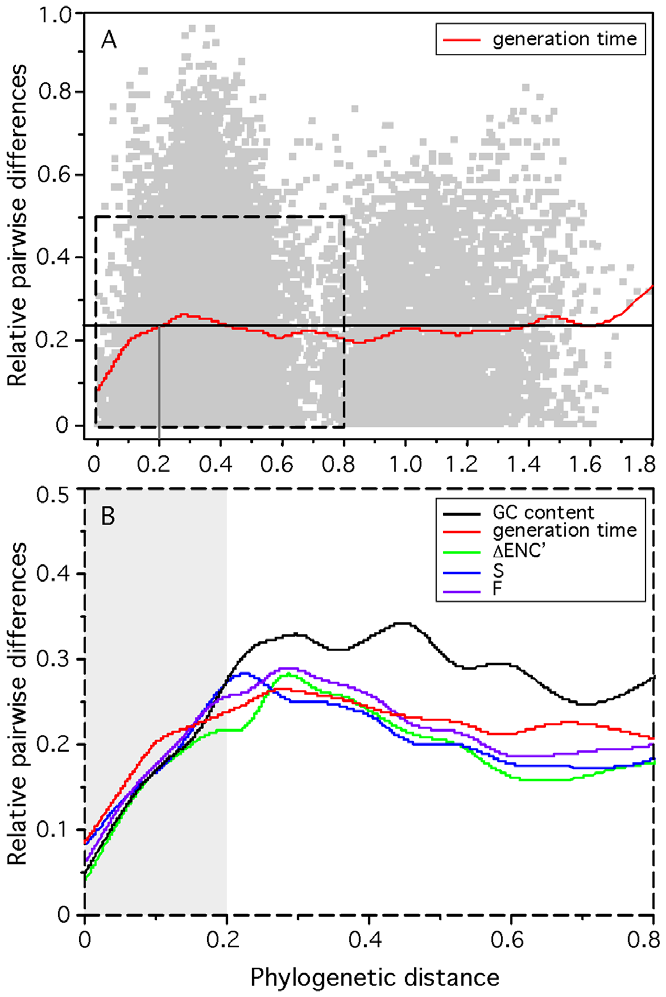
Relative difference between the minimum generation time, codon usage bias indices, and G+C content of pairs of organisms and their phylogenetic distance for 214 prokaryotes. Pairwise phylogenetic distances were computed from the matrix of the phylogenetic tree reconstruction (see Materials and Methods: phylogenetic analysis). Pairwise differences in doubling times (box-cox transform of d), codon usage bias indices ΔENC′, S and F and G+C content were normalized by the maximum observed difference in the 22791 pairs dataset (eq. 10). (A) The datapoints are represented in light gray. The red line represents a flexible spline fit (λ = 0.01). The black horizontal line represents the average relative pairwise difference. (B) The lines represent a flexible spline fit (λ = 0.01). For short distances (light gray area), the spearman correlations between phylogenetic distance and the relative difference in minimal generation times, ΔENC′, S and F and G+C content are respectively: 0.21, 0.28, 0.28, 0.29, 0.26 (all p-values<0.0001).

Phylogenetic dependencies between species may introduce a potentially important confounding factor in our analysis. If doubling times have important phylogenetic inertia then closely related genomes are bound to have similar growth rates and similarly important growth-related traits because their last common ancestor is too recent for these genomes to have diverged significantly. Hence, similarity in growth-related traits would not represent independent adaptive processes [50]. To test the effect of phylogenetic dependences we made an independent contrast analysis using a 16S-based phylogenetic tree (see Materials and Methods). All but one variable remained highly significantly correlated with minimal generation times after control for phylogenetic dependencies (Table 1). We have no explanation for the only exception, corresponding to the distance to the origin of replication of ubi-tRNA genes. We then analyzed how the difference in minimal generation times between two genomes increased with evolutionary distance (Figure 2A). This shows that when genomes are distant more than 0.2 substitutions/nt in our alignment there is no correlation between the two variables. Less than 8% of all pairs of genomes are distant by less than this threshold distance. This shows that evolutionary inertia on minimal growth rates is indeed low, often limited to the genera. We then performed the same analysis for all other variables (Figure 2B). This shows that even at low evolutionary distances, the minimal generation time has the lowest evolutionary inertia. It is thus tempting to speculate that changes in minimal growth rates tend to pre-date changes in growth-related traits, and not the other way around.

**Table 1:** Most informative attributes for the prediction of minimum generation time.

Variable	Individual ρ	Individual R^2^	Cumulative R^2^	Order	Ordered contribution R^2^
ΔENC′^A^	−0.70^++^	0.50^++/**^	0.50^++^	1	0.50^++^
S^A^	−0.60^++^	0.39^++/**^	0.56^++^	2	0.06^++^
rRNA position^B^	0.36^++^	0.15^++/**^	0.59^+^	3	0.03^+^
ubi-tRNA position^B^	0.41^++^	0.21^++/NS^	0.60	4	NS
rRNA number^C^	−0.66^++^	0.41^++/**^	0.61	5	NS
tRNA position^B^	0.35^++^	0.18^++/*^	0.61	6	NS
tRNA number^C^	−0.59^++^	0.33^++/**^	0.61	7	NS
ubi-tRNA number^C^	−0.68^++^	0.40^++/**^	0.61	8	NS
rpol position^B^	0.42^++^	0.18^++/**^	0.61	9	NS
rp position^B^	0.42^++^	0.17^++/**^	0.61	10	NS

A codon usage bias effects.

B replication-associated gene dosage effects.

C gene multiplicity effects.

NS: non-significant p-value; ^++^ p-value<0.001; ^+^ p-value<0.05.

After phylogenetic dependency correction: ^**^ p-value<0.001; ^*^ p-value<0.05; NS: non-significant p-value.

The results of a stepwise forward regression are given, where the most informative attributes enter first. Individual and cumulative coefficients of determination (R^2^) are given for the 10 genomic attributes under study. Individual and cumulative R^2^ are, respectively, the fraction of the variance of minimum generation time explained by the variable alone and by the variable combined with all the variables above in the table (N = 188). The p-values before and after phylogenetic dependency correction are given for the individual R^2^. Species with unknown origins of replication were excluded.

In summary, low minimal generation times are associated with the optimization of the translation machinery through: codon usage bias, an increased number of rRNA and tRNA gene copies by gene amplification, and the transient replication associated gene dosage of highly expressed genes under exponential growth. This information could be useful to reprogram growth rates in prokaryotes by synthetic biology approaches because modification of these traits should modify minimal generation times. Indeed, lower growth rates result from deletion of rRNA operons and from inversions decreasing gene dosage effects [19],[51]. Similarly, lower codon usage bias leads to lower growth rates in viruses [52]. Naturally, not all traits are equally easy to manipulate. While insertions of extra rRNA operons, e.g. using plasmids, are relatively straightforward, extensive changes in codon usage bias are only viable if the whole sequence is synthesized *in vitro*. This is now possible for viruses and even small bacterial genomes [53],[54].

### Codon usage bias is the best determinant of minimum generation time

Having delimited a range of 10 variables that correlate significantly with maximal growth rates (column *Individual R^2^* in Table 1), we estimated their predictive power using stepwise forward regressions. This allows to iteratively introduce in the model the most contributing variables while minimizing the number of variables in the model by excluding the ones without significant explanatory power [55]. For this analysis, we only used the 188 species for which we could retrieve an origin of replication (out of 214). To normalize the data we used a box-cox transformation Φ_λ_(d), which in this case approximates to the commonly used log-transformation (Figure S3). We focused on the increase in explained variance given by the inclusion of each variable (column *Cumulative R^2^* in Table 1). The highest contributing variables are ΔENC′, S and the relative distance of the rRNA genes to the origin of replication (*R^2^ contribution* column in Table 1). Prokaryotic genes often cluster in operons. We therefore tested if there were changes in the results if we had used operons instead of genes. We did this in the most significant positional variable, rDNA, and found no differences in the correlation with doubling time (ρ = 0.37 for genes and ρ = 0.36 for operons, both p-values<0.0001). Although rRNA operon multiplicity has a high individual explanatory power, it doesn't add new information into the model when codon usage bias, which has higher explanatory power, is already included. Hence, adaptation to fast growth is very strongly correlated in terms of gene multiplicity and codon usage bias, possibly because both are essentially associated with the optimization of translation. Genome organization around the origin of replication is less correlated with codon usage bias, possibly because it reflects the impact of replication rates on transcription: faster DNA polymerases lead to lower gene dosage effects for a similar generation time.

We then tested if the phylogenetic information could be a good predictor of minimal generation times. For this we made a stepwise regression where we added one more variable: the generation time of the most closely related genome. This variable adds little additional information (R^2^ = 0.65 versus R^2^ = 0.61 without the variable). The first variable to enter in the stepwise regression is still ΔENC′ (Table S3). This result is consistent with the abovementioned low phylogenetic inertia of minimal generation times. Since phylogenetic information is not as amenable to mechanistic interpretation as the other variables we didn't include it in the final predictor.

ΔENC′ and S both measure the intensity of selection for optimization of the translation of highly expressed genes. However, because they do it differently they both carry significant predictive power. These are the only genomic traits mentioned above that can be calculated from partial genome sequences, an undeniable advantage for the construction of a sequence-based predictor of minimum generation time. Evaluation of the codon usage bias does not require prior knowledge about the origin of replication, we can thus build our predictor on the full dataset (N = 214).

Since together ΔENC′ and S have larger explanatory power than individually (R^2^_ΔENC′_ = 0.44, R^2^_S_ = 0.33, R^2^_both_ = 0.49, p-value<0.0001), we combined them using principal component analysis. The first component, explaining 47% of the variance of minimum generation times, was called F (ρ = −0.66, p-value<0.0001). A preliminary linear predictor of Φ_λ_(d) in function of F was obtained by a least squares regression (N = 214, R^2^ = 0.47):
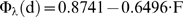
(1)


### Fast growth while coping with extreme temperatures

**Figure 3:**
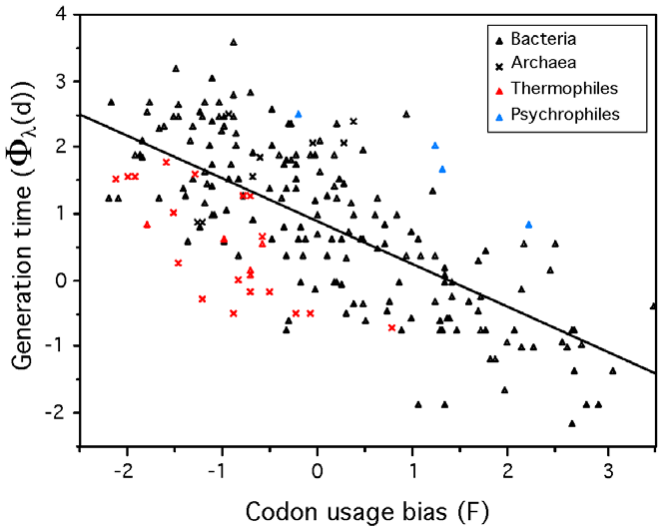
Minimum generation time (d) versus codon usage bias for 214 prokaryotes. F (first principal component of ΔENC′ and S) and Φ_λ_(d) (Box-Cox transform of d) are negatively correlated (ρ = −0.66). Line fitted by least squares regression: Φ_λ_(d) = 0.8741−0.6496 F (R^2^ = 0.47, p-value<0.0001).

**Figure 4:**
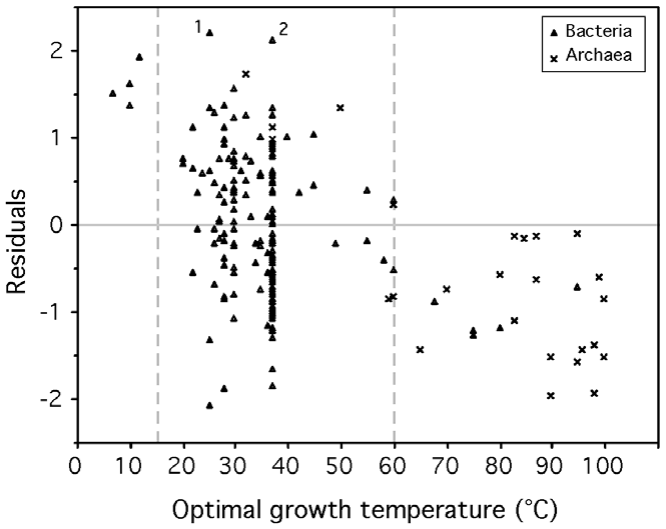
Correlation between the residuals of the model (eq. 1) and optimal growth temperature (OGT). Sperman correlation ρ = −0.37, p-value<0.0001. Residuals are positive for psychrophiles (OGT<15°C) and negative for thermophiles (OGT>60°C), indicating that for the former (latter) the observed minimal generation time is lower (higher) than expected from the genomic signatures. Relevant outliers: ^1^ *Sodalis glossinidius morsitans* and ^2^ *Mycobacterium leprae*.

The fit of the model showed that psychrophiles and thermophiles are systematically grouped above and below the prediction line, respectively (Figure 3). This suggests that part of the deviation from the model is biologically relevant and not a mere product of poor modeling or measurement errors. The residuals of the regression, representing the deviations to the model, are negatively correlated with optimal growth temperature (ρ = −0.37, p-value<0.0001, Figure 4). Naturally, we used minimal generation times obtained at optimal growth temperatures, therefore this result does not reflect slower growth at low temperatures of species with higher optimal growth temperature. This is also not an indication of higher growth rates at optimal growth temperatures in thermophiles. In fact, there is no significant difference of minimal generation times between thermophiles, mesophiles and psychrophiles (p-value>0.05 for ANOVA and Wilcoxon tests). This is also not caused by the over-representation of archaea among thermophiles, since archaea and bacteria do not have significantly different deviations to the model (p-value>0.1, Wilcoxon test). The association between deviations to the model and optimal growth temperature indicates that psychrophiles (thermophiles) are slower (faster) growers than expected given their genome growth-associated traits. While the above residuals are from a regression where only codon usage bias was used, we found similar patterns while analyzing the residuals of regressions using only information on gene multiplicity or replication associated gene dosage effects (data not shown). Hence, the association of deviations of the growth-related traits with optimal growth temperature is not exclusive to codon usage bias. Since there are no differences in minimal generation times between the different groups this suggests that for a given minimal generation time the psychrophiles require more structured genomes than mesophiles and these more than thermophiles.

Fast-growth associated traits are probably under weak selection, therefore subject to mutation-selection-drift balance. These results could then be interpreted as a sign of negative temperature dependence of selection for growth-related traits. At high temperature there would be less selection for optimization of these traits than at lower temperatures. Accordingly, mutations disrupting these traits are under strong purifying selection in psychrophiles and relaxed selection in thermophiles. For example, *Desulfotalea psychrophila*, *Methylobacillus flagellatus* and *Pyrococcus furiosus* present very similar genomic trends of adaptation to a minimum generation time of ∼3 hours (F = −0.23, −0.20 and −0.25 respectively). However, their respective observed minimum generation times are of 27, 2 and 0.6 hours for optimal growth temperatures of 7, 36 and 100°C.

The temperature dependence of the deviations to the model could also result from differences in effective population sizes in the different groups, if effective population size decreases with optimal growth temperature. We don't have data allowing the test of such a hypothesis. Instead, it is tempting to associate the effect of optimal growth temperature on the degree of genome optimization for fast growth with the dependence of enzymatic activity on temperature. At higher temperatures diffusion increases, water viscosity and activation energy decrease, facilitating rapid reactions [56] and could thus lead to lower requirements for growth-associated traits. As a case in point, psychrophiles have the highest multiplicity of rRNA and tRNA genes [57], whereas even fast-growing thermophiles have few copies, with a maximum of 4 rRNA operons in *Thermoanaerobacter tengcongensis* and *Carboxydothermus hydrogenoformans*. High temperatures possibly increase the catalytic rates of translation-associated reactions, and also the tRNA diffusion into ribosomes, allowing quick start and maintenance of exponential growth with fewer genes. This leads to weaker selection for gene multiplicity, lower codon usage bias and lower replication associated gene dosage effects. Hence, while we find no evidence that psychrophiles grow slower than other prokaryotes, they do show a tendency to strongly select for growth-related traits.

**Figure 5:**
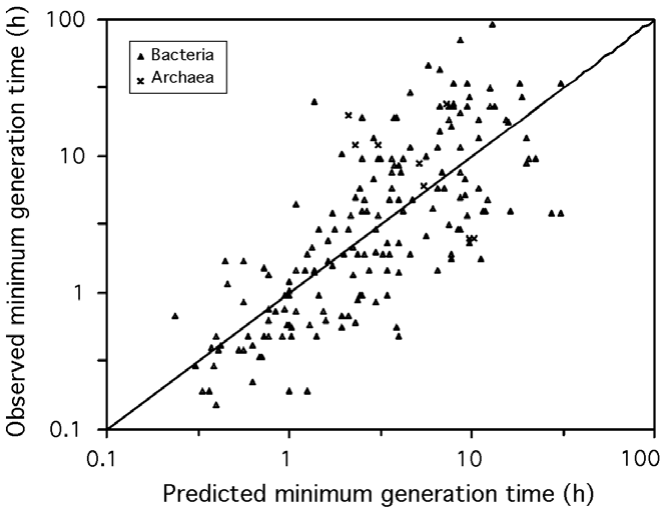
Observed versus predicted minimum generation time. The mesophilic predictor based on codon usage bias (eq. 3) was applied to the 187 mesophilic prokaryotic genomes. The diagonal black line corresponds to the identity.

After derivation, our predictor of minimal generation times (d in hours) (N = 214, R^2^ = 0.58) including optimal growth temperature (OGT in °C) becomes:
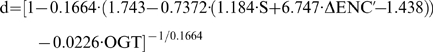
(2)See Materials and Methods for a detailed derivation of the equations. For mesophilic organisms this simplifies to (N = 187, R^2^ = 0.59, Figure 5):
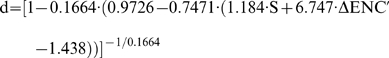
(3)We made a program to compute the expected minimal generation time given sequences of highly expressed genes and other genes in genomes. The program is publicly available at http://mobyle.pasteur.fr/cgi-bin/portal.py?form=growthpred. The information on ribosomal proteins for all the genomes and metagenomes used in this work can be found at the same site.

### Evolution of growth rate traits during genome reduction

We next investigated the genomes of mesophiles deviating most from the model. The highest positive residuals, corresponding to genomes with lower than expected maximal growth rates, are from the genomes of *Sodalis glossinidius morsitans* and *Mycobacterium leprae*, with observed generation times ∼18 and 35 times slower than expected. These genomes have the highest number of pseudo-genes within our data set (respectively, 49% and 50% of non-coding DNA), resulting from an ongoing process of genome reduction [58],[59]. It has been estimated that pseudogenes in *M. leprae* have an average age of ∼9 million years and have accumulated ∼15% of changes since then [60]. Naturally, synonymous positions of functional genes should evolve at least as slowly. Thus, even if selection for biased codon usage decreases, the slow pace of accumulation of synonymous substitutions by drift takes a long time to lower the bias down in highly expressed genes to the new value expected by the mutation-selection equilibrium for the new maximal growth rate. The lower phylogenetic inertia of minimal generation times, compared with other traits, namely codon usage bias (Figure 2B), justifies why the highest positive residues are among the genomes that have higher pseudogene density, in agreement with suggestions of a recent dramatic shift in lifestyle. Indeed, *S. glossinidius* and *M. leprae* grow much slower than the other closely related mycobacteria and free-living enterobacteria [61],[62]. Genomes that have endured slow growth for a long period of time such as *Buchnera aphidicola*, *Rickettsia typhi* or *Mycoplasma pneumoniae* have now lost any putative ancient organization related to high growth rates. These genomes thus conform to the predictions of maximal growth rates based on genome analysis.

### Prediction of growth rates from partial data

**Figure 6:**
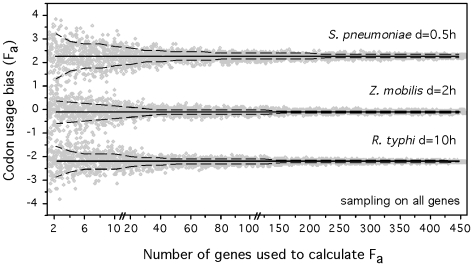
Accuracy of the determination of composite codon usage bias (F_a._) with varying sample size. F_a_ was calculated on randomly chosen samples (from 2 up to 450 genes) of all genes while using the full dataset of highly expressed genes. 100 iterations were effectuated for each sample size. The results for 3 organisms (one fast, slow and intermediate grower) are represented. The full black lines correspond to the whole genome value of F and the dashed lines to the standard deviations. Each data point is represented in gray.

We adapted the codon usage bias indices to make them computable from partial genomic and metagenomic data (see Materials and Methods). Measuring these variables on small sets of genes inevitably introduces some uncertainty in the estimation of the parameters. To evaluate the associated error, we sampled sets of genes of varying cardinality from mesophilic genomes for which we know the doubling time. We did this for non-highly expressed genes comparing them with the whole dataset of highly expressed genes (HEG), and inversely. The resulting ΔENC′ and S values were then subject to principal components analysis, of which the first component (F_a_) was compared with the one obtained from the whole genome. The results for 3 organisms (fast, slow and intermediate growers) are represented in Figure 6 for the first set of experiments and in Figure S4 for the latter. As expected, the estimates of F_a_ are less accurate with decreasing sample size. We then varied the sample size of both populations of genes and found that the analysis still had a remarkable power even when considering only 5 highly expressed and 5 non-highly expressed genes. In this case, a discrete classification of the mesophilic species (see Materials and Methods) into very fast, fast, intermediate and slow resulted in 50% exact classifications (expected 25%) and 89% approximate or exact classifications (prediction matching the same observed class or the adjacent ones, expected 59%). Even in this extremely small set of 10 genes, we only found 7% of slow growers predicted as fast or very fast or vice-versa (expected 29%). Therefore, a robust coarse qualitative assessment of minimal generation times can be made even with as few as ten genes (see Table S4 for a comparison of the results of discrete classification with the total set of genes, 40 genes, 20 genes and 10 genes). Such genome samples are easily accessible in metagenomic data from low diversity environments. For the other environments, the increase in coverage or the use of large-insert bacterial artificial chromosome libraries will also produce sufficiently large contigs [63].

### Prediction of growth rates in prokaryotic communities

Given the possibility of inferring minimum generation times from partial genomic data, we selected published metagenomic datasets to test 2 hypotheses: First, that environmental factors such as presence of toxic contaminants or resource availability influences the growth rate strategies of the resident microbial populations. Second, that fast growers are favored during the colonization phase of a new niche.

Environmental samples can be interpreted either as collections of pseudo-genomes or as metagenomes. In the former approach sequences putatively assigned to one same species can be put together in pseudo-genomes. In this approach, a large fraction of the data is lost because most species genomes are not sequenced and because genomes are so diverse in terms of gene repertoires that some genes will not match a template genome of the same species [64]. This approach has the advantage that if species are well known we can make more informed interpretations and we can control for phylogenetic dependencies. In the latter approach the sequences are all put together and treated as a great single meta-genome. This has the advantage of using all the data, including all the elusive non-cultivated prokaryotes, and accounts for the different availability of different species by their different quantitative contributions to the sample. However, it does not allow controlling for phylogenetic dependencies. We have preferred to use the second approach because we wanted to account for uncultivated species and relative frequencies of each species. We then confirm the results using the first approach.

#### Growth rate strategies in different environments

We first used our partial genomic data predictor on 3 datasets corresponding to very different environments for which simple predictions of maximal growth rates could be made: the human distal gut microbiome [65], the Waseca county farm soil metagenome [66] and the acid mine drainage biofilm metagenome [67] (for details of each dataset, see Tables S5 and S6). The human gut is a very rich environment, with periodic high nutrient inflow and with an important wash out rate to a poor outside environment. As a result, bacteria proliferating in the gut are subject to a feast-and-famine lifestyle, which has been proposed to select for very high growth rates [16],[68]. On the other extreme, the acid mine drainage biofilm reflects adaptation to a stable, nutrient poor and extremely toxic environment. In this situation one expects to find organisms that grow slowly but have great capacity to withstand stressful conditions [69]. The farm soil is an intermediate environment, where all the array of growth rates might be found, reflecting different life strategies (colonizers, stress resistant, capable competitors, etc) [70]. We therefore expected to find low average minimal generation times in the gut, intermediate in the soil and high in the acid mine drainage biofilm.

**Figure 7:**
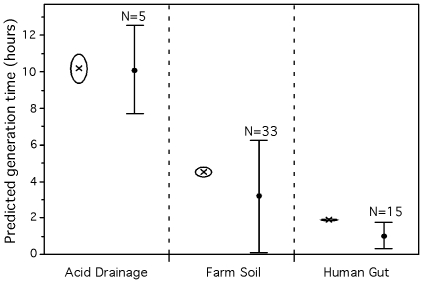
Average predicted minimum generation time for 3 environmental metagenomes. Crosses represent the average for the whole metagenome approach while dots represent the average for the pseudo-genome approach. All predictions were calculated with the predictor for mesophilic organisms (eq. 3). The average minimum generation time of the whole metagenome (crosses) and the respective standard deviation (open circles) were generated with 1,000 bootstraps on the dataset of all genes and highly expressed genes independently. The 3 whole-metagenome datasets are all significantly different (p-value<0.001). Minimum generation times were calculated using the whole genome of the sequenced genomes matching proteins of the metagenome (see Materials and Methods: classification of metagenomes into pseudo-genomes). The number of matching sequenced genomes are given above the average (dots) and standard deviation (bars) of the predictions. The 3 pseudo-genomes datasets are all significantly different (Tukey-Kramer: p-value<0.05).

Each metagenome was processed to obtain gene sequences large enough to allow meaningful measures of codon usage bias (see Materials and Methods). We then used the predictor for mesophiles (equation 3) to obtain average minimum generation times for each set. We found that the predicted average minimum generation times were of 1.8h (human gut), 4.6h (farm soil) and 10.2h (acid drainage) (Figure 7). These differences are highly significant as computed by bootstrap sampling on genes in the datasets (p-value<0.001 for details see Materials and Methods: Bootstrap on metagenomes). These samples were taken in environments with different temperatures. Since we showed that optimal growth temperature affects the predicted generation times, we repeated the analysis controlling for this effect. For this we used the average optimal growth temperature of the pseudo-genomes found in the sample (see below; 40°C for the acid mine, 30°C for the farm soil and 37°C for the human gut). The differences between the datasets remain significant after this control (p-value<0.001). This shows that our method gives results matching our expectations in that the human gut selects for fast-growers while toxic environments do not.

It is interesting to compare these growth rates with bacteria that are known to be part of these communities. In the human gut, clostridia, bacteroides and enterobacteria constitute a significant fraction of the community and by far the best studied one. Representative species such as *E. coli*, *E. faecalis*, *L. johnsonii* and *C. perfringens* have doubling times smaller than the community average of 1.8h (0.4h, 0.5h, 0.9h and 0.2h, respectively) and whole genome prediction of doubling times in conformity (0.8h, 0.7h, 0.6h and 0.4h). However, the predominant species in healthy adults, *Bacteroides thetaiotaomicron*, has an observed minimum generation time (1.5h) smaller than what is expected by its growth-associated genomic traits (3.4h). Again, typical soil bacteria, such as the *Streptomyces* or the alpha-proteobacteria, tend to have doubling times lower than the community average of 4.6h. Yet, they have generation times higher than the above-mentioned bacteria from the human gut, e.g. 2.2h for *Streptomyces coelicolor*, 2.4h for *Mesorhizobium loti* and 8h for *Nitrobacter winogradskyi*, with predictions 2.1h, 3.0h and 6.0h, respectively. In the acid mine drainage biofilm there are very few species, thus the scaffolds available correspond to almost complete genomes. These include two species for which generation times have been experimentally evaluated: *Ferroplasma acidarmanus* Type I with d = 4h [71] and *Leptospirillum* sp. Group II with d = 12h [72]. These observed values are close to the obtained by our predictions 6h and 13h respectively, using the scaffolds.

The lag between the growth rates of the best-studied bacteria of the human gut and farm soil and our metagenomics results could be due to a bias in our method. However, the analysis above shows that while smaller sequences reduce the accuracy of the estimates they do not seem to bias them in a given direction. It may thus be that the gap underlies a biological cause, the heterogeneity of these systems and the bias of cultivable organisms. Adhesion to the gut wall in biofilms and persistence in the soil under intense competition favors slower growth rates. Yet, cultivation methods will favor the isolation of fast growers. In order to further detail the metagenomic datasets in terms of the variance of its constituents, we classified the metagenomic proteins into pseudo-genomes (see Materials and Methods and details in Table S7). 11% of the proteins of the human gut microbiome matched 15 sequenced genomes, while 0.05% of the farm soil proteins matched 33 sequenced genomes. This shows that approaches based on aggregating sequences around pseudo-genomes ignore the majority of the data. Importantly, these results suggest a higher biodiversity in the farm soil than in the human gut, as expected, and it demonstrates that most of it is not represented in the sequenced genomes available to date. We then computed the predicted minimum doubling times for the matching sequenced genomes (using the whole genomes) and for the large scaffolds available for the 5 species present in the acid mine biofilm. The average minimal doubling times of the 3 environments are still significantly different (Tukey-Kramer: p-value<0.05). The results show that the human gut presents clearly the lowest variance in minimal doubling times. This is in agreement with a high selection pressure for fast growers in the human gut. On the other hand, the farm soil environment presents the highest variance (Figure 7), suggesting the coexistence of microorganisms with different life-strategies. Furthermore, as a control for possible phylogenetic dependencies, we repeated the analysis using one species per genera in each environment. The results remain significant (Tukey-Kramer: p-value<0.05). Hence, the pseudo-genome approach allows the analysis of the environment diversity in terms of growth rates and matches the expectation that highly toxic and very nutrient rich environments are less diverse in this respect.

#### Ecological succession in the human gut

**Figure 8:**
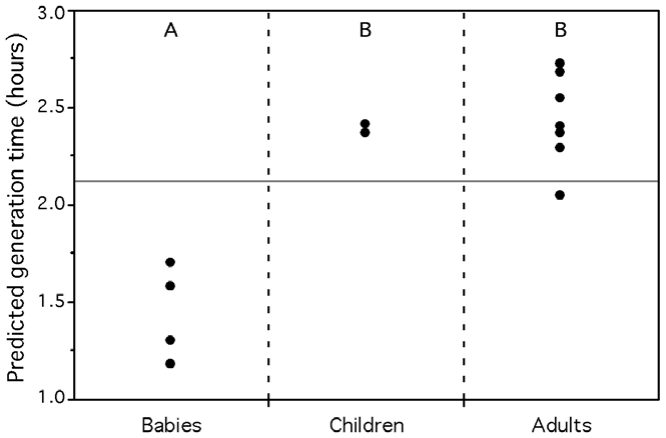
Average predicted minimum generation time for the gut metagenomes of humans of different age groups. Unweaned babies are 3, 4, 6, and 7 months old. Weaned children are 1.5 and 3 years old. Adults are between 24 and 45 years old. Groups not connected by the same letter (A or B) are significantly different (Tukey-Kramer: p-value<0.005). The full horizontal line represents the average of the predictions for all individuals.

The gastrointestinal tract of a healthy fetus *in utero* is sterile. Microorganisms from the mother and the surrounding environment are acquired during the birth process and thereafter through breast-feeding and social interaction. However, not all of them will succeed in colonizing the gastrointestinal tract. The gut microbial community is initially dominated by enterobacteria and streptococci, with subsequent establishment of the anaerobic *Bacteroides*, *Clostridium* and *Bifidobacterium*. The latter clearly dominating for the entire breast-feeding period [73]. As solid diet is introduced, a more complex and dense gut ecosystem will develop and eventually reach a dynamical balance with its host. The first phase of this microbial succession, corresponding to the colonization of the nutrient-rich gut, should be dominated by faster growing organisms. This corresponds to the classical prediction in evolutionary ecology that colonizers are in general fast-growers [74]. To test it, we used our partial genomic data predictor on the gut microbiome of several adults, weaned children and unweaned babies [75]. The latter represent the niche under colonization. Indeed the gut metagenome of unweaned babies (prediction 1.4h) have significantly lower average minimum generation time than those of children (2.4h) and adults (2.4h) (ANOVA: R^2^ = 0.84, p-value<0.0001, Figure 8). The results are identical for adults and young children (1.5 and 3 years old), which suggests a rapid evolution of the gut microflora after diet alteration.

It is also interesting to notice the difference between the Japanese and American gut metagenomes. No Bacteroides 16S rRNA genes were found in the American dataset, although other studies confirm that Bacteroides are predominant in the human gut microbiota [76],[77]. This discrepancy was identified by the authors and attributed to possible complications in the DNA extractions [65]. However, such complex protocols might induce other sampling biases, harder to detect. Thus, one must be cautious when comparing metagenomes from different projects/laboratories. Nevertheless, the average minimum generation times of the 4 Japanese babies remain significantly lower than those of the two American adults (ANOVA: R^2^ = 0.70, p-value = 0.02).

We also classified the metagenomic proteins of the Japanese gut microbiomes into pseudo-genomes and calculated predicted minimum generation times based on the matching whole genome sequences. 11% of the proteins of the gut microbiomes matched sequenced genomes. The average number of species found in the different age groups' microbiota are not significantly different (26 for babies, 26 for children and 22 for adults, Wilcoxon: p-value = 0.64). We analyzed the groups' doubling times in 3 different ways, in order to compare them to the results of the whole metagenome analysis (Table S8). First, there is no significant difference in the arithmetic average of the predictions for the babies, children and adults. However, if we weight the contribution of the value of each pseudo-genome by the number of proteins of the metagenome that matched it, then we recover a result close to the one of the whole metagenome, where unweaned babies have a significantly lower average minimal generation time than children and adults (Tukey-Kramer: p-value<0.01). We find similar results when keeping only one species per genera (Table S8). Therefore, we find no evidence that the gut microbiota of adults is composed of slower growing species than the one of babies. Instead, the relative abundance of species in the gut population accounts for faster growing communities in babies. This highlights the interest of the whole metagenome approach, which intrinsically takes into account this information.

### Concluding remarks

Our results show that minimal generation times imprint genome organization and sequence of Bacteria and Archaea. They also show that such information allows the prediction of maximal growth rates from sequence alone. Naturally, organisms rarely grow at maximal growth rates because they rarely meet ideal growth conditions. As a result, our data does not allow predicting growth rates in specific environments. Yet, information on the maximal growth rates coupled with biochemical modeling can eventually lead to prediction of growth rates in particular media [78]. The optimization of growth related traits allows the quick start of exponential growth upon favorable environmental changes and allows faster growth also under sub-optimal conditions. In this sense, maximal growth rates are proxies of the capacity of the species to rapidly produce biomass, to quickly change growth rates and to take advantage of rich media. If such traits were not important, then random mutations erasing codon usage bias, genome organization and gene multiplicity would not be selected against and none of these traits would be found. Instead, we have shown that the majority of genomic traits that correlate significantly with maximal growth rates are also strongly correlated among themselves. This is a consequence of a shared selective pressure leading to the adaptation of the cellular machinery for high growth potential.

We found that some unexpected variables have strong influence in genome optimization for growth, notably ongoing genome reduction and optimal growth temperature. The slow pace of substitutions is likely to explain the higher than expected codon usage bias in reducing genomes. The association of optimal growth temperature with deviations to expected growth rates might result from the enzymatic rate dependence on temperature, but that remains openly speculative until comparative data on the translation biochemistry of psychrophiles and thermophiles becomes available. Other variables may also influence maximal growth rates and genome optimization. We detail three types. First, while we made exhaustive searches in primary literature to collect minimal generation times, there is substantial incertitude on these. We may have missed some publications with lower generation times, but more importantly, current growth conditions are still far from optimal for many prokaryotes. This introduces a bias in the analysis, since slow-growers are much less studied than fast-growers. For example, a search in the PubMed database of the number of articles citing each of the species we analyzed showed that this number is highly correlated with the minimal generation times (ρ = −0.45, p-value<0.0001). Hopefully, our data will be of use to pinpoint the species for which a revision of growth times will be most likely to be fruitful, since the largest residuals that are not explained by temperature or ongoing genome reduction might concern prokaryotes for which generation times are less accurate. Secondly, other measures of within genome bias in gene expression such as strength of ribosome binding sites, promoters, operon organization and genome structure might improve our predictor [79]–[82]. Yet, since our 10 growth-associated traits were all highly correlated, increasing the number of growth-associated traits in the analysis is unlikely to add much information. Thirdly, environmental variables that can affect growth can have more important, and for the moment unforeseeable roles, especially if they affect enzymatic activity. As the database grows larger we will be able to better pinpoint them by systematic analysis of deviations from the predicted values, as we found for optimal growth temperature.

Along the discussion of our results we have systematically interpreted deviations from the model in a selective perspective. This is based on the extensive literature showing the physiological effects of selection for growth-related traits in exponentially growing cells. Yet, most growth related traits, e.g. codon usage bias, are expected to be under weak selection thus liable to genetic drift depending on the effective population size (Ne). If Ne is independent of maximal growth rates this will only result in increased variance in our predictor. But if Ne is negatively correlated with minimal generation times then fastest growing organisms could have more growth-related traits than slow growers because of higher selection coefficient for these traits and/or because of more efficient selection, ie higher Ne. In this case, our predictor for growth rates would also be a predictor of effective population size. While selection for growth related traits is not under dispute, systematic deviations from the model could be strongly influenced by the effective population size. For example, if Ne were negatively correlated with optimal growth temperature it might explain the deviations we observe. Unfortunately, we have no way of systematically computing Ne for our sample of prokaryotes. Lynch [83] computed Ne.u, where u is the mutation rate, for 11 bacteria, all mesophiles. Assuming similar mutation rates the 3 slowest growing bacteria are in the 4 top positions. The highest Ne is for *Prochorococcus marinus*, by far the slowest-growing bacteria in the set and thought to be one of the most abundant species on earth [84]. Also of relevance, the recent application of a model for predicting trophic lifestyle to marine metagenomic data has shown that copiotrophs dominate free-living microbial populations [85]. These results suggest that among free-living bacteria slow-growing species tend to outnumber fast-growing ones. On the other hand, highly reduced symbiotic genomes, supposedly with very low Ne, tend to have high minimal generation times, with some exceptions among Mollicutes. These contradictory trends suggest no obvious correlation between growth rates and effective population size. There is also little evidence for a correlation between maximal growth rates and absolute population sizes. This is because population sizes result from average, not maximal, growth rates and are moderated by the rates of cell death. While most free-living slow-growers lack growth-related traits because they do not endure selection for fast growth, it is possible that bacteria with sudden contractions of population sizes will endure a degradation of growth-related traits leading to lower growth rates. The availability of population data for a growing number of genomes will hopefully allow understanding the evolution of growth-related traits in a population genetics framework.

Besides contributing to the understanding of genome evolution at different maximal growth rates, our results open two important avenues of further research. First, we find that a composite index of codon usage bias allows for the accurate prediction of the type of growth expected from a given prokaryote. Surprisingly, this can be done even with very few genes paving the way for the understanding of a key physiological parameter from partial sequence data alone. This will be of use in the incoming surge of metagenomic data that contains sequences of species about which we ignore everything. Aggregation of metagenomic data into phylotypes will also allow analyzing the diversity of communities in terms of minimal generation times. Second, our data will also be useful in the delineation of experiments aiming at increasing or lowering growth rates in synthetic biology. The production of many metabolites of industrial interest is in conflict with the cell capacity to replicate. Our results point some ways in which prokaryotes can be engineered to grow slower, e.g. by decrease in codon usage in ribosomal proteins, deletion of rRNA operons or ubi-tRNAs. If it is of interest to maximize the production rate of biomass, then inverse interventions, conjugated with experimental evolution, may significantly accelerate the pace at which a lineage acquires the capacity to grow faster. It would be naïve to think that just changing rRNA expression will necessarily result in higher growth rates. In fact, slow growing bacteria often show higher than needed ribosome concentrations [86],[87]. To change growth rates one probably needs to use design growth-related traits optimized genomes and then use experimental evolution to select for high growth rates in environments more favorable to growth than the natural one. Our work, by ranking the information provided by the different traits, provides guidelines for the relevance of each trait in such design. Third, the proposed predictor of minimum generation times applied to metagenomic datasets allows testing central theories in microbial ecology associated with growth rates. Metagenomic datasets give a unique access to whole microbial communities, regardless of their cultivability. As metagenomics develops, longer scaffolds will be available, with enough information to predict the growth rate of the corresponding species. Also, key genomes for specific niches are being sequenced, with example of the Human Microbiome Project sequencing 1000 microbial reference genomes. The emergence of all this new material will open new avenues of research in microbial ecology and evolution.

## Materials and Methods

### Whole genome data

We retrieved 214 genome sequences, 1 per species, from GenBank Genomes (ftp://ftp.ncbi.nih.gov/genomes/Bacteria/). Genes were extracted from annotation data and pseudo-genes were ignored. Genes of the transcription/translation machinery (RNA polymerase, rRNAs, ribosomal proteins) were identified by the annotation fields, or, when not possible, by homology from the genomes of closely related species. A pair of genes were regarded as orthologous if they were reciprocal best hits with more than 40% sequence similarity and less than 20% difference in protein length, as measured by a end-gaps free sequence alignment. tRNAs were searched with tRNAscanSE [88] using the default parameters for bacteria or archaea. When the tRNA anticodon matched a previously published list of nearly ubiquitous tRNAs [35] it was included in the list of ubi-tRNAs. Optimal growth temperatures (OGT) were retrieved for 204 of the 214 organisms from the DSMZ database (http://www.dsmz.de/microorganisms/). Psychrophiles and thermophiles were defined as organisms whose OGT is under 15°C and over 60°C, respectively. We extracted from primary literature the minimal generation times (d) for the 214 species of bacteria and archaea (Table S1).

### Metagenomic data

The contigs from the 3 metagenomic datasets used in Figure 7 were retrieved from GenBank (http://www.ncbi.nlm.nih.gov/books/bv.fcgi?rid=metagenomics), including the acid mine drainage biofilm (AADL01000001–AADL01002534), the Waseca County Farm Soil (AAFX01000001–AAFX01139340), and the human distal gut microbiome (AAQK01000001–AAQK01010488, AAQL01000001–AAQL01012020). The contigs from the 13 healthy humans gut microbiomes of the Human Metagenome Consortium Japan (HMGJ; http://www.metagenome.jp/) were also retrieved from GenBank under the following accession numbers: subject F1-S (BAAU01000001–BAAU01028900), subject F1-T (BAAV01000001–BAAV01036326), subject F1-U (BAAW01000001–BAAW01016539), subject F2-V (BAAX01000001–BAAX01036455), subject F2-W (BAAY01000001–BAAY01030198), subject F2-X (BAAZ01000001–BAAZ01031237), subject F2-Y (BABA01000001–BABA01035177), subject In-A (BABB01000001–BABB01020226), subject In-B (BABC01000001–BABC01009958), subject In-D (BABD01000001–BABD01037296), subject In-E (BABE01000001–BABE01020532), subject In-M (BABF01000001–BABF01016164) and subject In-R (BABG01000001–BABG01034797).

### Distance to the origin of replication

Predicted origins of replication were retrieved from DoriC database (http://tubic.tju.edu.cn/doric/) [89]. Archaea often have multiple and difficult to assess origins of replication [90]. Therefore, archaea were excluded from the calculation of distances to the origin of replication and subsequent correlations to growth rate.

Relative distance to the origin of replication is calculated as the smallest circular distance of the gene to the origin of replication divided by half of the chromosome size. Hence, 0 corresponds to the origin of replication, 0.5 to half the replicon and 1 to the position opposite to the origin, typically the terminus.

### Codon usage bias

We used two different measures to assess the difference in codon usage biases between the average and the highly expressed genes: the ΔENC′ and the S indices.

ΔENC′ is an empirical estimator of the strength of selection acting on codon usage bias in highly expressed genes [35]. For each genome, the ENC′ value [91] was calculated separately for the concatenation of all the coding sequences (ENC′_all_) and for the concatenation of the ribosomal protein genes (ENC′_rib_), using the average coding nucleotide frequency. The ΔENC′ was then calculated as:
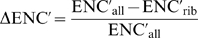
(4)S is also an estimator of the strength of selection acting on codon usage bias, but based on the mutation-selection balance between pairs of codons, where one is fitter. Following Sharp, we compute S using the frequency of codons for four amino acids: Phe (C_1_ = UUC, C_2_ = UUU), Ile (C_1_ = AUC, C_2_ = AUU), Tyr (C_1_ = UAC, C_2_ = UAU), Asn (C_1_ = AAC, C_2_ = AAU). Codons C_1_ and C_2_ are recognized by the same tRNA. By Watson-Crick rules, the codon-anticodon interaction between C_1_ and the anticodon is better. Hence, C_1_ should be favored in genes having translation-associated codon usage bias. For each of the 4 amino acids mentioned above, we calculated the frequency of the optimal codon P = C_1_/(C_1_+C_2_) in all proteins (P_all_) and in ribosomal proteins (P_rib_). The S component for each amino acid is then given by:
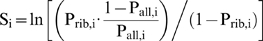
(5)S is the weighted mean of the *S_i_* values [46].

As alternatives to ΔENC′ and S, we also tested the use of the genome ENC′ and of ribosomal proteins ENC′. The former was a very bad predictor of growth rates (R^2^ = 0.12), the latter was as good predictor as ΔENC′ (respectively R^2^ = 0.53 and R^2^ = 0.54, for the mesophiles), but correlated with the genome G+C content, suggesting that while the genome ENC′ has little informative power it calibrates for compositional biases when it's included in the computation of ΔENC′.

### Codon usage bias adapted to metagenomic data

Both ΔENC′ and S calculations were adapted to use gene-level information (ΔENC′_a_ and S_a_) instead of the genomic-level information (concatenation of the genes as previously done). When analyzing metagenomes, concatenating all of the sequences would erroneously increase the mean effective number of codons (ENC′) of the dataset, because each organism might have a different codon usage bias (i.e. a different set of preferred codons). This is not the case for the calculation of S [46], which only takes into account the codon usage for 4 amino acids, for which the optimal codon is the same in all species. The problem of analyzing a mixture a sequences from different species can be circumvented if ENC′ is calculated gene by gene.

Thus, we calculated for each gene separately, ENC′ and P (P = C_1_/C_1_+C_2_, for the 4 amino acids indistinctly) (C_1_ and C_2_ codons are listed above in the ‘Codon usage bias’ section). Then, we calculate the average ENC′ and P for the set of genes coding for ribosomal proteins and for the all the genes separately (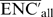 and 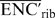, 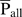 and 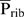). Afterwards, we compute ΔENC′_a_ and S_a_ using:
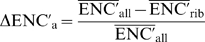
(6)

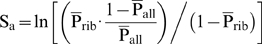
(7)AWK and R scripts and C source of the programs to compute ENC′ and P calculations for each gene are available from the authors.

For the set of all genes, open reading frames (ORFs) with a minimum size of 450bp were retrieved using EMBOSS function getorf. For the set of highly expressed genes, ribosomal proteins were retrieved by similarity with a database of ribosomal proteins of all sequenced genomes available to date (e-value<10^−5^).

### Bootstrap on metagenomes

The error bars of average growth rates of environmental metagenomes correspond to the standard deviation of the predictions generated with 1000 bootstraps on the metagenome dataset of all genes and highly expressed genes independently and simultaneously. In order to compare the average predicted minimal doubling time of different metagenomes, we computed the difference between the predictions of pairs of environments, for each bootstrap iteration. The significance (p-value) of the comparison of averages of different metagenomes was calculated as the proportion of the differences that didn't match the expectation. For example, for the acid mine (AM) and the farm soil (FS), we calculated for each iteration d_AM_-d_FS_. The acid mine's average doubling time is larger than the farm soil. The significance of this difference has a p-value p‰ if one finds p out of 1000 iterations where d_AM_-d_FS_<0 (e.g. 10 iterations<0 give a p-value = 0.01). If no such iteration is found we mark p<0.001.

### Discrete classification

The observed minimum generation times (d) of the mesophilic species were discretized into four classes: very fast (d<1h, N = 46), fast (1h<d<2h, N = 26), intermediate (2h<d<5h, N = 41) and slow (d≥5h, N = 74). The predicted continuous values for the 187 species were obtained with the mesophilic predictor, using 5 highly expressed genes and 5 other genes (both randomly chosen in the complete sets, 1000 random experiments). These were discretized in the same way and compared to the observed ones. The accuracy of the classification was evaluated from the proportion of exact, approximate and wrong classifications (%), respectively defined as the proportion of 1) predictions matching the same observed class, 2) predictions matching the same observed class or the adjacent ones (e.g. predicted ‘fast’ when actually ‘very fast’) and 3) slow growers predicted as fast or very fast and inversely.

### Box-Cox transformations

The Box-Cox power transformation aims at ensuring that the usual assumptions for linear models hold [55]. We used it to linearize the relation between minimum generation time (d) and the other variables. For example, in the association between d and F, a Box-Cox transformation was applied to d:
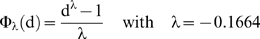
(8)


### Principal component analysis

In order to retrieve the most relevant information of ΔENC′ and S combined, a PCA was performed and the first principal component, which was highly correlated to growth rate, was named F.
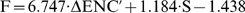
(9)


### Derivation of the predictor

By linear regression, the following relation between the transformation of minimum generation time (eq. 8) and the first principal component (F) of codon usage bias indices ΔENC′ and S (eq. 9):
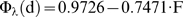
Replacing F (eq. 9), we obtain:
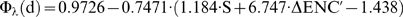
Reversing the transformation of minimum generation time (eq. 8), we obtain our predictor (eq. 3):
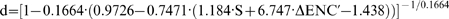



### Phylogenetic analysis

We build a phylogenetic tree using the 16S rDNA subunit for each species. We made a multiple alignment of the 16S sequences with MUSCLE [92], followed by manual correction with SEAVIEW [93]. The tree was computed by maximum likelihood with PHYML [94] using the model HKY+Γ(4)+I. Pairwise phylogenetic distances were computed from the distance matrix. Phylogenetic contrast analysis was done with the ape package in R using generalized estimation equations (GEE) [95].

### Pairwise relative differences

Pairwise differences of minimum doubling time Δd were calculated for the 214 prokaryotes. The difference of the box-cox transforms of doubling times for the pair of species were normalized by the maximum observed difference in the 22791 pairs.
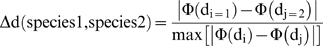
(10)The relative pairwise differences in codon usage bias indices ΔENC′, S, F and G+C content were calculated the same way, for the 188 prokaryotes with known origins of replication.

### Classification of metagenomes into pseudo-genomes

We mapped each protein of a given metagenome dataset in a given template genome. Template genomes were taken among 601 completely sequenced genomes. For each species we chose one single strain to avoid statistical bias. By default we used the first published strain. Mapping was done as follows: 1) for each protein of the metagenome dataset we find highly similar homologues within every proteome using quickhit, a companion of swelfe [96], that allows to quickly find highly similar protein sequences. 2) The hits were then aligned using exact end-gap free Needleman-Wunsch alignments. 3) A given protein was added to one, and only one, pseudo-genome if it matched the corresponding template genome, if this was the best among all matches and if the protein similarity was higher than 95%.

## Supporting Information

Figure S1 Genomic signatures correlated to minimum generation time (d) for 214 prokaryotes. Negative correlation between d and the number of (A) rRNA operons, (B) tRNA genes, (C) ubiquitous tRNA genes, in the genome. (D) Non-significant correlation between d and the number of non-ubiquitous tRNA genes in the genome. Spearman correlations are given (ρ) with p-values<0.0001 for (A–C) and p-value = 0.06 for (D).
(0.11 MB TIF)

Figure S2 Genomic signatures correlated to minimum generation time (d) for 188 bacteria. Positive correlation between d and the relative distance from the origin of replication to (A) RNA polymerase genes, (B) tRNA genes, (C) ribosomal protein coding genes, (D) ubiquitous tRNA genes. Spearman correlations are given (ρ) with all p-values<0.0001. Species with unknown origins of replication were excluded.
(0.11 MB TIF)

Figure S3 The box-cox transformation Φ_λ_(d) used to normalize our data versus the decimal logarithm. The transformations were plotted for a minimum generation time (d) of the range of our dataset: 0.16h to 240h.
(0.01 MB TIF)

Figure S4 Accuracy in the determination of composite codon usage bias (F_a_) with varying sample size. F_a_ was calculated on a randomly chosen sample (from 2 up to 36 genes) of highly expressed genes while using the whole dataset of control genes. 100 iterations were effectuated for each sample size. The results for 3 organisms (fast, slow and intermediate growers) are represented. The full black lines correspond to the whole genome value of F and the dashed lines to the standard deviations. Each data point is represented in gray.
(0.07 MB TIF)

Table S1 List of the 214 genomes composing our dataset and their characteristics. Generation times were retrieved from the literature. We defined the minimum generation time (Column “d”) as the smallest value reported (Column “d reference”) for one species. For very few bacteria the generation times for closely related species were used. The optimum growth temperature of the species (Column “OGT”) was retrieved from DSMZ database. The predicted origin of replication (Column “Ori”) was retrieved from DoriC database.
(0.56 MB DOC)

Table S2 List of ubiquitous tRNAs (ubi-tRNA) in 102 bacterial species, previously published [35].
(0.04 MB DOC)

Table S3 Most informative attributes for minimum generation time prediction. The results of a stepwise forward regression are given, where the most informative attributes enter first. Individual and cumulative coefficients of determination (R^2^) are given for the 10 genomic attributes under study and one extra attribute: the minimum generation time of the closest organism in our 16S phylogenetic tree. Individual and cumulative R^2^ are, respectively, the fraction of the variance of d explained by the variable alone and by the variable combined with all the variables above in the table (N = 188). The p-values before and after phylogenetic dependency correction are given for the individual R^2^. Species with unknown origins of replication were excluded.
(0.04 MB DOC)

Table S4 Accuracy of a discrete classification of the 187 mesophilic species. Classification into 4 classes: very fast (d<1h, N = 46), fast (1h<d<2h, N = 26), intermediate (2h<d<5h, N = 41) and slow (d≥5h, N = 74). Proportion of exact, approximate and wrong classifications (%), respectively defined as the proportion of 1) predictions matching the same observed class, 2) predictions matching the same observed class or the adjacent ones (e.g. predicted ‘fast’ when actually ‘very fast’) and 3) slow growers predicted as fast or very fast and inversely. Genes were chosen randomly in the complete subsets (ribosomal proteins (HEG) or other proteins (non-HEG)) for 1000 random experiments.
(0.03 MB DOC)

Table S5 Description of the metagenomes of the 3 environmental samples.
(0.03 MB DOC)

Table S6 Description of the human gut metagenomes for 3 age groups.
(0.03 MB DOC)

Table S7 List of the sequenced complete genomes matching the proteins of the environmental metagenomes.
(0.04 MB DOC)

Table S8 Comparison of whole metagenome and pseudo-genome analysis for the 3 age groups human gut metagenomes.
(0.03 MB DOC)
