# Supplementary figures and images for: The Systemic Imprint of Growth and Its Uses in Ecological (Meta)Genomics
Source: PLoS Genet. 2010 Jan 15;6(1):e1000808. doi: 10.1371/journal.pgen.1000808 (PMC2797632; doi:10.1371/journal.pgen.1000808)

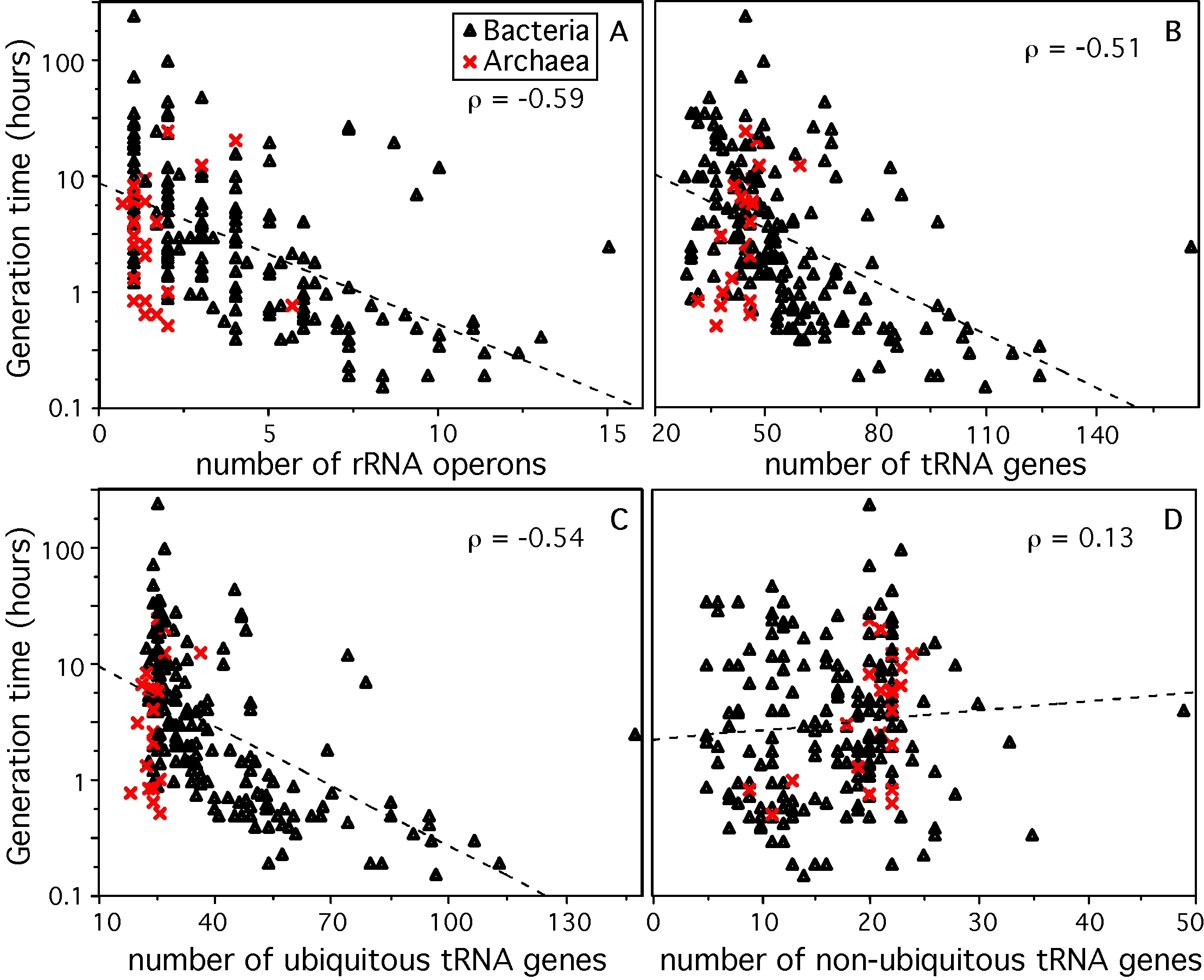

Supplement: Figure S1 — Genomic signatures correlated to minimum generation time (d) for 214 prokaryotes. Negative correlation between d and the number of (A) rRNA operons, (B) tRNA genes, (C) ubiquitous tRNA genes, in the genome. (D) Non-significant correlation between d and the number of non-ubiquitous tRNA genes in the genome. Spearman correlations are given (ρ) with p-values<0.0001 for (A–C) and p-value = 0.06 for (D). (0.11 MB TIF) [file pgen.1000808.s001.tif]

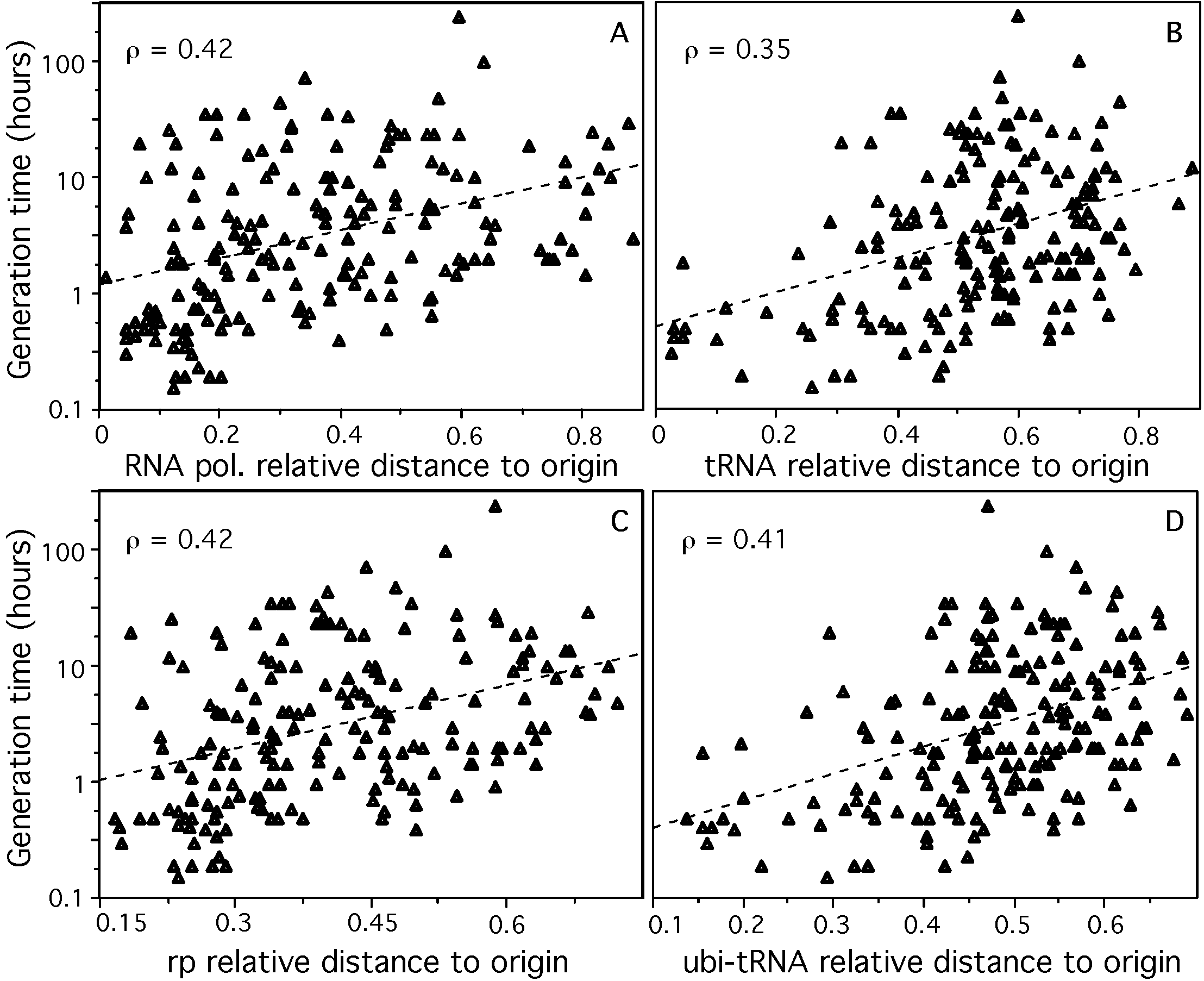

Supplement: Figure S2 — Genomic signatures correlated to minimum generation time (d) for 188 bacteria. Positive correlation between d and the relative distance from the origin of replication to (A) RNA polymerase genes, (B) tRNA genes, (C) ribosomal protein coding genes, (D) ubiquitous tRNA genes. Spearman correlations are given (ρ) with all p-values<0.0001. Species with unknown origins of replication were excluded. (0.11 MB TIF) [file pgen.1000808.s002.tif]

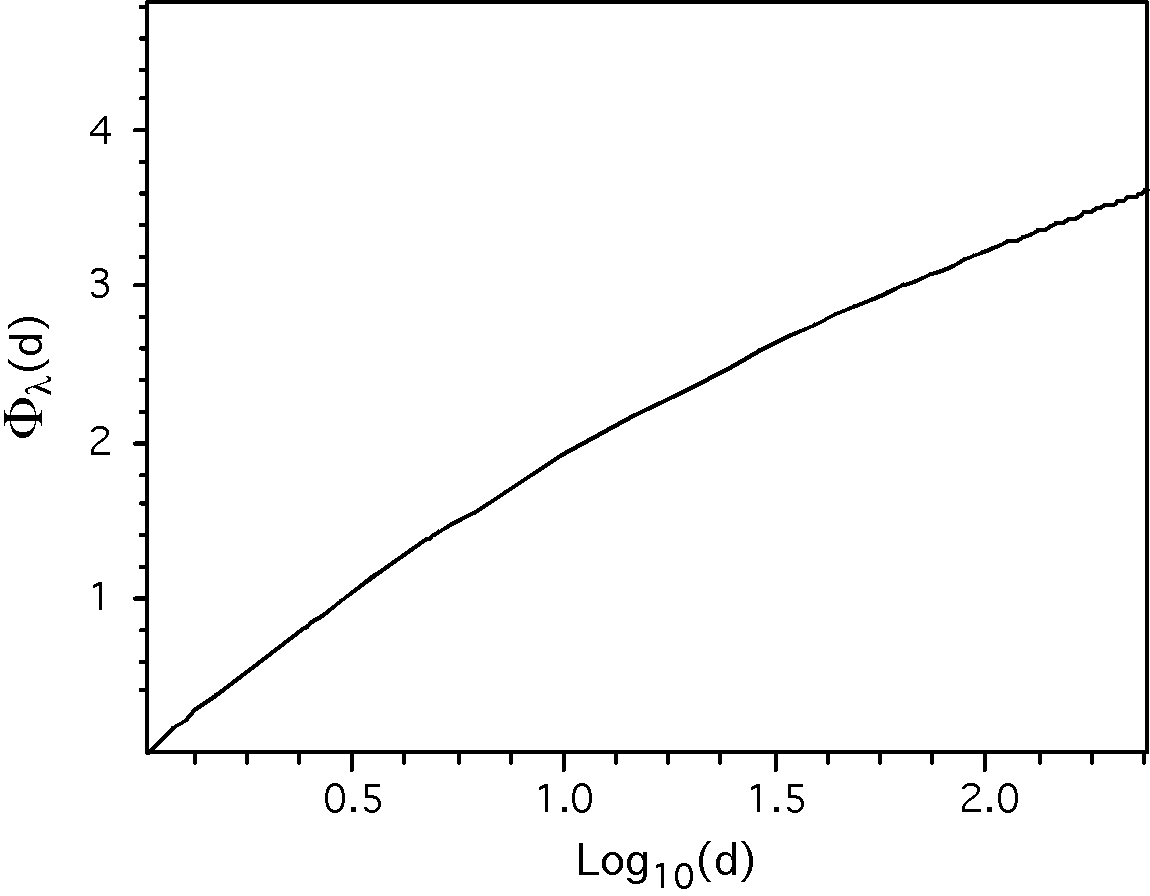

Supplement: Figure S3 — The box-cox transformation Φλ(d) used to normalize our data versus the decimal logarithm. The transformations were plotted for a minimum generation time (d) of the range of our dataset: 0.16h to 240h. (0.01 MB TIF) [file pgen.1000808.s003.tif]

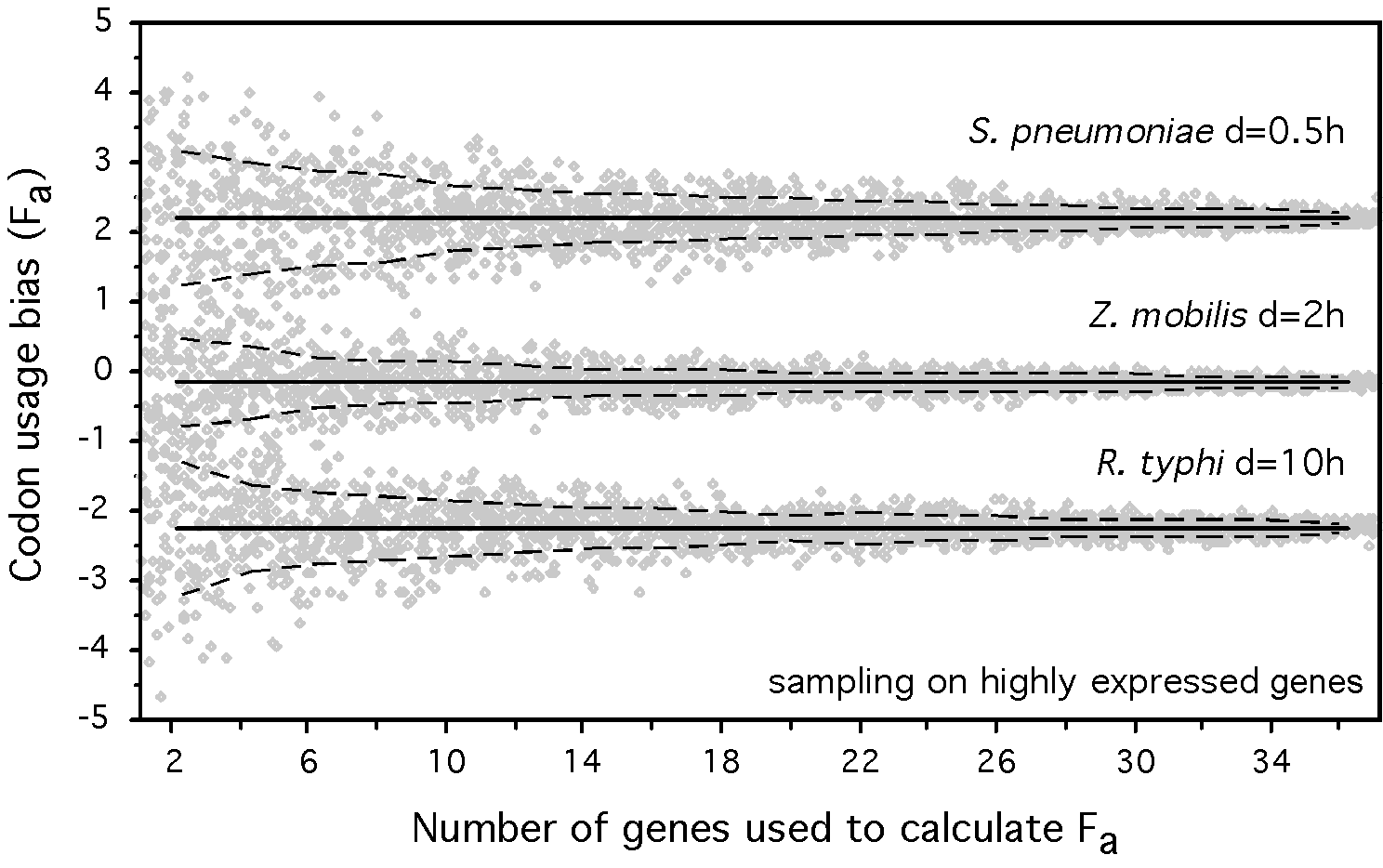

Supplement: Figure S4 — Accuracy in the determination of composite codon usage bias (Fa) with varying sample size. Fa was calculated on a randomly chosen sample (from 2 up to 36 genes) of highly expressed genes while using the whole dataset of control genes. 100 iterations were effectuated for each sample size. The results for 3 organisms (fast, slow and intermediate growers) are represented. The full black lines correspond to the whole genome value of F and the dashed lines to the standard deviations. Each data point is represented in gray. (0.07 MB TIF) [file pgen.1000808.s004.tif]
