# Supplementary material for: The Systemic Imprint of Growth and Its Uses in Ecological (Meta)Genomics
Source: PLoS Genet. 2010 Jan 15;6(1):e1000808. doi: 10.1371/journal.pgen.1000808 (PMC2797632; doi:10.1371/journal.pgen.1000808)
Supplement: Table S1 — List of the 214 genomes composing our dataset and their characteristics. Generation times were retrieved from the literature. We defined the minimum generation time (Column “d”) as the smallest value reported (Column “d reference”) for one species. For very few bacteria the generation times for closely related species were used. The optimum growth temperature of the species (Column “OGT”) was retrieved from DSMZ database. The predicted origin of replication (Column “Ori”) was retrieved from DoriC database. (0.56 MB DOC) [file pgen.1000808.s005.doc]

**Supplementary Table 1**: **List of the 214 genomes composing our dataset and their characteristics**. Generation times were retrieved from the literature. We defined the minimum generation time (Column “d” in hours) as the smallest value reported (Column “d reference”) for one species. For very few bacteria the generation times for closely related species were used. The optimum growth temperature of the species (Column “OGT” in °C) was retrieved from DSMZ database. The predicted origin of replication (Column “Ori”) was retrieved from DoriC database.

| **Species name** | **Ori** | **OGT** | **d (h)** | **d reference** |
| --- | --- | --- | --- | --- |
| Acidovorax avenae | 27627 | 28 | 11 | [1] |
| Acaryochloris marina | 3125510 | 25 | 45 | [2] |
| Actinobacillus pleuropneumoniae | 1883252 | 37 | 0.75 | [3] |
| Acinetobacter ADP1 | 3598158 | 30 | 0.5 | [4] |
| Aeromonas hydrophila | 4729112 | 28 | 0.35 | [5] |
| Aeropyrum pernix |  | 95 | 4 | [6] |
| Agrobacterium tumefaciens C58 | 2841174 | 26 | 3 | [7] |
| Alcanivorax borkumensis | 3119635 | 28 | 10 | [8] |
| Anaeromyxobacter dehalogenans | 1396 | 30 | 9.2 | [9] |
| Anaplasma marginale | 1147944 | 37 | 21.6 | [10] |
| Anaplasma phagocytophilum | 20557 | 37 | 7 | [11] |
| Anabaena variabilis | 1502 | 20 | 8 | [12] |
| Aquifex aeolicus | 209842 | 95 | 1.8 | [13] |
| Arthrobacter aurescens | 1420 | 30 | 2 | [14] |
| Arcobacter butzleri | 2341023 | 37 | 0.66 | [15] |
| Archaeoglobus fulgidus |  | 85 | 4 | [16] |
| Azoarcus sp EbN1 | 1677877 | 28 | 4.3 | [17] |
| Bacillus anthracis Ames | 1748 | 37 | 0.5 | [18] |
| Bacillus cereus ATCC 14579 | 1622 | 30 | 0.3 | [19] |
| Bacteroides fragilis | 110151 | 37 | 0.63 | [20] |
| Bacillus halodurans | 4202339 | 30 | 0.6 | [21] |
| Bartonella henselae Houston-1 | 1930403 | 37 | 3 | [22] |
| Bacillus licheniformis ATCC14580 | 1848 | 37 | 0.58 | [23] |
| Bartonella quintana Toulouse | 1580795 | 37 | 3 | [24] |
| Bacillus subtilis 168 | 1751 | 37 | 0.43 | [25] |
| Bacteroides thetaiotaomicron VPI5482 | 4171767 | 37 | 1.47 | [26] |
| Bacillus thuringiensis konkukian 97-27 | 1750 | 30 | 0.42 | [27] |
| Bdellovibrio bacteriovorus | 1417 | 30 | 1.4 | [28] |
| Bifidobacterium longum NCC2705 | 1625766 | 37 | 1.51 | [29] |
| Bordetella bronchiseptica RB50 | 5337389 | 37 | 1.42 | [30] |
| Borrelia burgdorferi | 458037 | 30 | 4 | [31] |
| Borrelia garinii PBi | 460244 | 37 | 4 | [31] |
| Bordetella pertussis 12822 | 4084466 | 37 | 3.8 | [32] |
| Brucella abortus biovar1 9-941 | 2010519 | 37 | 2 | [33] |
| Bradyrhizobium japonicum USDA110 | 680553 | 26 | 20 | [34] |
| Brucella melitensis 16M | 2116944 | 37 | 2 | [35] |
| Brucella suis 1330 | 1993857 | 37 | 2 | [36] |
| Buchnera aphidicola Sg | 641022 | 22 | 36 | [37] |
| Buchnera aphidicola Baizongia pistaciae | 1 | 22 | 36 | [37] |
| Burkholderia mallei ATCC23344 | 3006500 | 37 | 0.75 | Personal communication |
| Burkholderia pseudomallei K96243 | 2376 | 37 | 1 | Personal communication |
| Candidatus Blochmannia floridanus | 704749 | 30 | 36 | [38] |
| Caulobacter crescentus | 4016703 | 30 | 1.5 | [39] |
| Carboxydothermus hydrogenoformans | 2399734 | 68 | 2 | [16] |
| Campylobacter jejuni | 1324 | 37 | 1.5 | [40] |
| Caldivirga maquilingensis |  | 83 | 8 | [41] |
| Candidatus Pelagibacter ubique | 335453 |  | 30 | [42] |
| Chlamydophila abortus S26/3 | 1144232 | 37 | 24 | [43] |
| Chloroflexus aurantiacus | 5258006 | 49 | 6 | [44] |
| Chlamydophila caviae GPIC | 1173153 | 37 | 24 | [43] |
| Chlamydia trachomatis M | 1072919 | 37 | 24 | [43] |
| Chlamydia pneumonia A | 1 | 37 | 24 | [43] |
| Chlorobium tepidum TLS | 1127 | 30 | 2 | [45] |
| Chlamydia trachomatis | 719974 | 37 | 24 | [43] |
| Chromobacterium violaceum ATCC12472 | 236386 | 26 | 0.8 | [46] |
| Clostridium acetobutylicum ATCC824 | 1808 | 37 | 0.58 | [47] |
| Clostridium perfringens 13 | 1784 | 37 | 0.2 | [48] |
| Clostridium tetani E88 | 50965 | 37 | 0.5 | [49] |
| Coxiella burnetii RSA 493 | 1835517 | 37 | 8 | [50] |
| Corynebacterium glutamicum | 1576 | 30 | 1.2 | [51] |
| Colwellia psychrerythraea | 5367414 | 10 | 7.14 | [52] |
| Delftia acidovorans | 26889 | 30 | 4.62 | [53] |
| Dehalococcoides ethenogenes 195 | 1599 | 35 | 19 | [54] |
| Desulfotalea psychrophila LSv54 | 709557 | 7 | 27 | [55] |
| Deinococcus radiodurans chromosome1 | 1183 | 30 | 1.5 | [56] |
| Desulfotomaculum reducens | 1691 | 37 | 20 | [57] |
| Desulfovibrio vulgaris vulgaris Hildenborough | 3570715 | 32 | 14 | [58] |
| Dinoroseobacter shibae | 3633375 | 33 | 4.08 | [59] |
| Ehrlichia canis | 11395 | 37 | 28 | [60] |
| Ehrlichia chaffeensis | 26939 | 37 | 19 | [60] |
| Enterococcus faecalis V583 | 1403 | 37 | 0.5 | [61] |
| Enterobacter sakazakii | 3960616 | 37 | 0.23 | [62] |
| Erwinia carotovora atroseptica SCRI1043 | 5063660 | 28 | 0.2 | [63] |
| Escherichia coli MG1655 | 3923657 | 37 | 0.35 | [64] |
| Francisella tularensis | 1892387 | 37 | 3 | [65] |
| Fusobacterium nucleatum | 641869 | 37 | 0.72 | [20] |
| Geobacter sulfurreducens PCA | 1368 | 30 | 6 | [66] |
| Gluconobacter oxydans 621H | 1139728 | 28 | 0.94 | [67] |
| Gloeobacter violaceus | 3438638 |  | 72 | [68] |
| Gramella forsetii | 3458688 |  | 4.17 | [69] |
| Haemophilus ducreyi 35000HP | 1698708 | 36 | 1.8 | [70] |
| Haemophilus influenzae | 603005 | 36 | 0.5 | [64] |
| Haloarcula marismortui |  | 50 | 12 | [71] |
| Halobacterium sp. Strain NRC-1 |  | 37 | 9 | [72] |
| Haloquadratum walsbyi |  | 37 | 24 | [73] |
| Herpetosiphon aurantiacus | 6345664 | 30 | 20 | [74] |
| Helicobacter hepaticus ATCC51449 | 1081990 | 37 | 4.2 | [75] |
| Helicobacter pylori | 1608998 | 37 | 2.4 | [76] |
| Hyperthermus butylicus |  | 99 | 2 | [16] |
| Ignicoccus hospitalis |  | 90 | 0.83 | [77] |
| Lactobacillus acidophilus NCFM | 1993119 | 45 | 1.8 | [78] |
| Lactobacillus johnsonii NCC533 | 1992003 | 30 | 0.9 | [79] |
| Lactococcus lactis IL1403 | 1726 | 40 | 0.7 | [80] |
| Lactobacillus plantarum WCFS1 | 1369 | 30 | 1.6 | [81] |
| Leptospira interrogans serovar lai 56601 chr1 | 1566 | 29 | 9 | [82] |
| Legionella pneumophila pneumophila Philadelphia 1 | 3397647 | 37 | 3.3 | [83] |
| Leifsonia xyli xyli CTCB07 | 1647 | 23 | 5 | [84] |
| Listeria innocua Clip11262 | 1675 | 22 | 0.6 | [85] |
| Listeria monocytogenes strain EGD | 1674 | 23 | 1 | [64] |
| Mannheimia succiniciproducens MBEL55E | 2281135 | 37 | 0.6 | [86] |
| Methanosarcina barkeri |  | 37 | 12 | [87] |
| Methanococcoides burtonii |  | 32 | 20 | [88] |
| Methylococcus capsulatus | 3302726 | 37 | 1.87 | [89] |
| Methylobacterium extorquens | 5470388 | 28 | 4.2 | [90] |
| Methylobacillus flagellatus | 1459 | 36 | 2 | [91] |
| Methanococcus jannaschii |  | 83 | 0.5 | [16] |
| Methanopyrus kandleri |  | 98 | 0.83 | [92] |
| Mesorhizobium loti | 4478943 | 26 | 2.4 | [93] |
| Methanobacterium thermoautotrophicum |  | 65 | 1 | [94] |
| Moorella thermoacetica | 228 | 58 | 5 | [95] |
| Mycobacterium avium paratuberculosis k10 | 1531 | 37 | 10 | [96] |
| Mycobacterium bovis bovis AF2122/97 | 1525 | 37 | 23 | [97] |
| Mycoplasma capricolum | 1009729 | 37 | 2.5 | [98] |
| Mycoplasma gallisepticum R | 2682 | 37 | 1 | Personal communication |
| Mycoplasma genitalium | 578582 | 37 | 12 | [99] |
| Mycoplasma hyopneumoniae 232 | 513000 | 37 | 2 | [100] |
| Mycobacterium leprae | 1567 | 37 | 240 | [101] |
| Mycoplasma mobile 163K | 1 | 20 | 10 | [102] |
| Mycoplasma mycoides mycoides SC | 1211535 | 45 | 2.2 | [98] |
| Mycoplasma pneumoniae | 816339 | 37 | 6 | [99] |
| Mycoplasma pulmonis UAB CTIP | 1 | 37 | 1.5 | [103] |
| Mycobacterium tuberculosis | 1525 | 37 | 19 | [97] |
| Myxococcus xanthus | 9139603 | 30 | 5 | [104] |
| Nanoarchaeum equitans |  | 90 | 0.75 | [105] |
| Neisseria gonorrhoeae FA1090 | 1917745 | 36 | 0.58 | [106] |
| Neisseria meningitidis A | 219873 | 37 | 0.72 | [107] |
| Nitrosomonas europaea ATCC19718 | 1591 | 27 | 18.5 | [43] |
| Nitrobacter winogradskyi | 114297 | 28 | 8 | [108] |
| Nocardia farcinica | 1990 | 37 | 3 | [109] |
| Nostoc sp. PCC 7120 | 2404398 |  | 12 | [110] |
| Oenococcus oeni | 1780070 | 24 | 10.5 | [111] |
| Paracoccus denitrificans | 2851745 | 30 | 2.1 | [112] |
| Pasteurella multocida PM70 | 1677118 | 37 | 1 | [113] |
| Parachlamydia sp UWE25 | 1 |  | 48 | [114] |
| Petrotoga mobilis | 1423266 | 55 | 12 | [115] |
| Photorhabdus luminescens laumondii TTO1 | 44490 | 28 | 0.5 | Personal communication |
| Photobacterium profundum SS9 | 4085022 | 10 | 2.5 | [116] |
| Pirellula sp | 5455000 | 28 | 10 | [117] |
| Picrophilus torridus DSM 9790 |  | 60 | 6 | [118] |
| Porphyromonas gingivalis W83 | 2342805 | 37 | 2.7 | [119] |
| Propionibacterium acnes KPA171202 | 1748 | 31 | 5.1 | [120] |
| Prochlorococcus marinus marinus CCMP1375 | 1750905 |  | 17 | [121] |
| Pseudomonas aeruginosa PA01 | 6264361 | 37 | 0.5 | [64] |
| Pseudoalteromonas haloplanktis | 3208603 | 26 | 0.5 | [122] |
| Psychromonas ingrahamii | 4558259 | 12 | 12 | [123] |
| Pseudomonas putida KT2440 | 8947 | 27 | 1.1 | [124] |
| Pseudomonas syringae tomato DC3000 | 6396882 | 27 | 1.47 | [125] |
| Pyrococcus abyssi |  | 96 | 0.62 | [16] |
| Pyrobaculum aerophilum |  | 100 | 3 | [16] |
| Pyrobaculum arsenaticum |  | 95 | 1.3 | [126] |
| Pyrococcus furiosus |  | 100 | 0.62 | [16] |
| Pyrococcus horikoshii |  | 98 | 0.62 | [16] |
| Ralstonia solanacearum GMI1000 | 3716277 | 30 | 4 | [127] |
| Rhodoferax ferrireducens | 40520 | 25 | 8 | [128] |
| Rhodopseudomonas palustris | 322962 | 33 | 9 | [129] |
| Rhodococcus sp. Strain RHA1 | 3871702 | 30 | 5.3 | [130] |
| Rhodospirillum rubrum | 4154190 | 28 | 3.8 | [131] |
| Rhodobacter sphaeroides | 3004887 | 32 | 3 | [132] |
| Rickettsia conorii Malish 7 | 1268361 | 35 | 4.1 | [133] |
| Rickettsia prowazekii | 1111140 | 35 | 10 | [134] |
| Rickettsia typhi wilmington | 1111114 | 35 | 10 | [135] |
| Rubrobacter xylanophilus | 1355 | 60 | 3.85 | [136] |
| Salinibacter ruber | 12594 | 42 | 14 | [137] |
| Salmonella typhimurium LT2 | 4083788 | 37 | 0.4 | [64] |
| Shewanella oneidensis MR-1 | 6419 | 30 | 0.66 | [138] |
| Sinorhizobium meliloti 1021 | 1 | 28 | 1.5 | [139] |
| Silicibacter pomeroyi | 4105854 |  | 1.65 | [140] |
| Sorangium cellulosum | 11354923 | 30 | 16 | [104] |
| Sodalis glossinidius | 4127547 | 25 | 26 | [141] |
| Sphingopyxis alaskensis | 1535 |  | 2.39 | [142] |
| Streptococcus agalactiae 2603VR | 1464 | 37 | 1.8 | [143] |
| Staphylococcus aureus strain N315 | 1879 | 34 | 0.4 | [144] |
| Streptomyces coelicolor A3(2) | 4269844 | 30 | 2.2 | [145] |
| Staphylococcus epidermidis ATCC 12228 | 1718 | 34 | 0.8 | [146] |
| Staphylothermus marinus |  | 87 | 6.3 | [147] |
| Streptococcus mutans UA159 | 1553 | 37 | 0.75 | [148] |
| Streptococcus pneumoniae TIGR4 | 1559 | 37 | 0.5 | [149] |
| Streptococcus pyogenes M1 | 1588 | 37 | 0.4 | [150] |
| Streptococcus thermophilus LMG18311 | 1551 | 37 | 0.43 | [151] |
| Sulfolobus acidocaldarius |  | 70 | 5.67 | [16] |
| Sulfolobus solfataricus |  | 87 | 6 | [16] |
| Sulfolobus tokodaii |  | 80 | 6 | [16] |
| Syntrophus aciditrophicus | 1529 | 35 | 24 | [152] |
| Synechococcus elongatus PCC6301 | 1620819 | 32 | 6.1 | [153] |
| Syntrophobacter fumaroxidans | 3503182 | 37 | 97.86 | [154] |
| Synechocystis PCC6803 | 665000 | 35 | 12 | [155] |
| Symbiobacterium thermophilum | 1378 | 60 | 4.2 | [156] |
| Synechococcus sp WH8102 | 2434168 | 32 | 6 | [157] |
| Thermoplasma acidophilum |  | 59 | 2.5 | [16] |
| Thiomicrospira crunogena | 2427456 | 30 | 1 | [158] |
| Thermosynechococcus elongatus BP-1 | 2338968 | 55 | 5.4 | [159] |
| Thermotoga maritima | 156961 | 80 | 1.2 | [160] |
| Thermoanaerobacter tengcongensis | 1697 | 75 | 1.1 | [161] |
| Thermus thermophilus HB27 | 1524341 | 75 | 2.5 | [162] |
| Thermoplasma volcanium |  | 60 | 2.5 | [16] |
| Treponema denticola ATCC35405 | 265084 | 37 | 5 | [163] |
| Trichodesmium erythraeum | 2409 | 28 | 25 | [164] |
| Treponema pallidum | 1399 | 37 | 33 | [165] |
| Tropheryma whipplei Twist | 1438 | 37 | 28 | [166] |
| Ureaplasma urealyticum | 42000 | 37 | 0.9 | Personal communication |
| Vibrio cholerae | 2961047 | 37 | 0.2 | [38] |
| Vibrio fischeri ES114 | 2906069 | 30 | 0.3 | [167] |
| Vibrio parahaemolyticus | 3288455 | 25 | 0.2 | [168] |
| Vibrio vulnificus CMCP6 chr1 | 1012335 | 25 | 0.16 | [38] |
| Wigglesworthia glossinidia brevipalpis | 697376 |  | 36 | Personal communication |
| Wolbachia endosymbiont TRS Brugia malayi | 1079740 |  | 14 | [169] |
| Wolinella succinogenes | 2110228 | 37 | 1 | [170] |
| Xanthomonas axonopodis | 1371 | 27 | 7 | [171] |
| Xanthomonas campestris | 1371 | 37 | 3 | [172] |
| Xanthomonas oryzae oryzae KACC10331 | 1374 | 28 | 2 | [173] |
| Xylella fastidiosa 9a5c | 1463 | 28 | 5.13 | [174] |
| Yersinia pestis CO92 | 4653618 | 37 | 1.25 | [175] |
| Yersinia pseudotuberculosis | 4744561 | 37 | 0.5 | [176] |
| Zymomonas mobilis | 2055615 | 27 | 2 | [177] |

**Supplementary Table 1 References:**

1. Song B, Palleroni NJ, Haggblom MM (2000) Isolation and characterization of diverse halobenzoate-degrading denitrifying bacteria from soils and sediments. Appl Environ Microbiol 66: 3446-3453.

2. Swingley WD, Hohmann-Marriott MF, Le Olson T, Blankenship RE (2005) Effect of iron on growth and ultrastructure of Acaryochloris marina. Appl Environ Microbiol 71: 8606-8610.

3. Fuller TE, Shea RJ, Thacker BJ, Mulks MH (1999) Identification of in vivo induced genes in Actinobacillus pleuropneumoniae. Microb Pathog 27: 311-327.

4. Parke D, Garcia MA, Ornston LN (2001) Cloning and genetic characterization of dca genes required for beta-oxidation of straight-chain dicarboxylic acids in Acinetobacter sp. strain ADP1. Appl Environ Microbiol 67: 4817-4827.

5. Hudson JA (1993) Effect of Preincubation Temperature on the Lag Time of Aeromonas-Hydrophila. Letters in Applied Microbiology 16: 274-276.

6. Robinson NP, Bell SD (2007) Extrachromosomal element capture and the evolution of multiple replication origins in archaeal chromosomes. Proc Natl Acad Sci U S A 104: 5806-5811.

7. Bell CR (1990) Growth of Agrobacterium tumefaciens under octopine limitation in chemostats. Appl Environ Microbiol 56: 1775-1781.

8. Yakimov MM, Timmis KN, Golyshin PN (2007) Obligate oil-degrading marine bacteria. Curr Opin Biotechnol 18: 257-266.

9. He Q, Sanford RA (2003) Characterization of Fe(III) reduction by chlororespiring Anaeromyxobacter dehalogenans. Appl Environ Microbiol 69: 2712-2718.

10. Gale KR, Leatch G, DeVos AJ, Jorgensen WK (1996) Anaplasma marginale: effect of challenge of cattle with varying doses of infected erythrocytes. Int J Parasitol 26: 1417-1420.

11. Wang X, Rikihisa Y, Lai TH, Kumagai Y, Zhi N, et al. (2004) Rapid sequential changeover of expressed p44 genes during the acute phase of Anaplasma phagocytophilum infection in horses. Infect Immun 72: 6852-6859.

12. Haury JF, Spiller H (1981) Fructose uptake and influence on growth of and nitrogen fixation by Anabaena variabilis. J Bacteriol 147: 227-235.

13. Guiral M, Tron P, Aubert C, Gloter A, Iobbi-Nivol C, et al. (2005) A membrane-bound multienzyme, hydrogen-oxidizing, and sulfur-reducing complex from the hyperthermophilic bacterium Aquifex aeolicus. J Biol Chem 280: 42004-42015.

14. Strong LC, Rosendahl C, Johnson G, Sadowsky MJ, Wackett LP (2002) Arthrobacter aurescens TC1 metabolizes diverse s-triazine ring compounds. Appl Environ Microbiol 68: 5973-5980.

15. Hilton CL, Mackey BM, Hargreaves AJ, Forsythe SJ (2001) The recovery of Arcobacter butzleri NCTC 12481 from various temperature treatments. Journal of Applied Microbiology 91: 929-932.

16. Torarinsson E, Klenk HP, Garrett RA (2005) Divergent transcriptional and translational signals in Archaea. Environmental Microbiology 7: 47-54.

17. Chee-Sanford JC, Frost JW, Fries MR, Zhou J, Tiedje JM (1996) Evidence for acetyl coenzyme A and cinnamoyl coenzyme A in the anaerobic toluene mineralization pathway in Azoarcus tolulyticus Tol-4. Appl Environ Microbiol 62: 964-973.

18. Chakrabarty K, Wu W, Booth JL, Duggan ES, Coggeshall KM, et al. (2006) Bacillus anthracis spores stimulate cytokine and chemokine innate immune responses in human alveolar macrophages through multiple mitogen-activated protein kinase pathways. Infect Immun 74: 4430-4438.

19. Olmez HK, Aran N (2005) Modeling the growth kinetics of Bacillus cereus as a function of temperature, pH, sodium lactate and sodium chloride concentrations. Int J Food Microbiol 98: 135-143.

20. Mangels JI, Lindberg LH, Vosti KL (1978) Quantitative evaluation of three commercial blood culture media for growth of anaerobic organisms. J Clin Microbiol 7: 59-62.

21. Sturr MG, Guffanti AA, Krulwich TA (1994) Growth and bioenergetics of alkaliphilic Bacillus firmus OF4 in continuous culture at high pH. J Bacteriol 176: 3111-3116.

22. Chenoweth MR, Somerville GA, Krause DC, O'Reilly KL, Gherardini FC (2004) Growth characteristics of Bartonella henselae in a novel liquid medium: primary isolation, growth-phase-dependent phage induction, and metabolic studies. Appl Environ Microbiol 70: 656-663.

23. van Dijk-Salkinoja MS, Planta RJ (1971) Rate of ribosome production in Bacillus licheniformis. J Bacteriol 105: 20-27.

24. Huang KY (1967) Metabolic Activity of the Trench Fever Rickettsia, Rickettsia quintana. J Bacteriol 93: 853-859.

25. Yoshikawa H, O'Sullivan A, Sueoka N (1964) Sequential replication of the Bacillus subtilis chromosome. III. Regulation of initiation. Proc Natl Acad Sci U S A 52: 973-980.

26. Anderson KL, Salyers AA (1989) Genetic evidence that outer membrane binding of starch is required for starch utilization by Bacteroides thetaiotaomicron. J Bacteriol 171: 3199-3204.

27. Kashyap S, Amla DV (2007) Characterisation of Bacillus thuringiensis Kurstaki strains by toxicity, plasmid profiles and numerical analysis of their cryIA genes. African Journal of Biotechnology 6: 1821-1827.

28. Pritchard MA, Langley D, Rittenberg S (1975) Effects of methotrexate on intraperiplasmic and axenic growth of Bdellovibrio bacteriovorus. J Bacteriol 121: 1131-1136.

29. Garro MS, Aguirre L, de Giori GS (2006) Biological activity of Bifidobacterium longum in response to environmental pH. Applied Microbiology and Biotechnology 70: 612-617.

30. Yuk MH, Harvill ET, Miller JF (1998) The BvgAS virulence control system regulates type III secretion in Bordetella bronchiseptica. Molecular Microbiology 28: 945-959.

31. De Silva AM, Fikrig E (1995) Growth and migration of Borrelia burgdorferi in Ixodes ticks during blood feeding. Am J Trop Med Hyg 53: 397-404.

32. Frohlich BT, Clark ERD, Siber GR, Swartz RW (1995) Improved Pertussis Toxin Production by Bordetella-Pertussis through Adjusting the Growth Media Ionic Composition. Journal of Biotechnology 39: 205-219.

33. Hoover DL, Friedlander AM (2005) Brucellosis. Textbook of Military Medicine: Medical Aspects of Chemical and Biological Warfare: The Virtual Naval Hospital Project. pp. 513-521.

34. Lopez-Garcia SL, Vazquez TE, Favelukes G, Lodeiro AR (2001) Improved soybean root association of N-starved Bradyrhizobium japonicum. J Bacteriol 183: 7241-7252.

35. Gallot-Lavallee T, Zygmunt MS, Cloeckaert A, Bezard G, Dubray G (1995) Growth phase-dependent variations in the outer membrane protein profile of Brucella melitensis. Res Microbiol 146: 227-236.

36. Ekaza E, Guilloteau L, Teyssier J, Liautard JP, Kohler S (2000) Functional analysis of the ClpATPase ClpA of Brucella suis, and persistence of a knockout mutant in BALB/c mice. Microbiology 146: 1605-1616.

37. Baumann P, Baumann L, Lai CH, Rouhbakhsh D (1995) Genetics, physiology, and evolutionary relationships of the genus Buchnera: intracellular symbionts of aphids. Annu Rev Microbiol 49: 55-94.

38. Holt JG (1984) Bergey's Manual of Systematic Bacteriology: Gram-negative Bacteria of general, medical, or industrial importance

Gram-negative Bacteria of general, medical, or industrial importance

1. Baltimore: Williams & WilkinsHolt, J.G.

39. Iba H, Fukuda A, Okada Y (1977) Chromosome replication in Caulobacter crescentus growing in a nutrient broth. J Bacteriol 129: 1192-1197.

40. Rollins DM, Coolbaugh JC, Walker RI, Weiss E (1983) Biphasic culture system for rapid Campylobacter cultivation. Appl Environ Microbiol 45: 284-289.

41. Itoh T, Suzuki K, Sanchez PC, Nakase T (1999) Caldivirga maquilingensis gen. nov., sp. nov., a new genus of rod-shaped crenarchaeote isolated from a hot spring in the Philippines. Int J Syst Bacteriol 49 Pt 3: 1157-1163.

42. Tripp HJ (2007) Genomic-assisted determination of the natural nutrient requirements of the cosmopolitan marine bacterium 'Candidatus Pelagibacter ubique'. PhD thesis Oregon State University.

43. Holt JG (1989) Bergey's Manual of Systematic Bacteriology: Archaeobacteria, Cyanobacteria, and remaining Gram-negative Bacteria

3. Baltimore: Williams & WilkinsHolt, J.G.

44. Pierson BK, Keith LM, Leovy JG (1984) Isolation of pigmentation mutants of the green filamentous photosynthetic bacterium Chloroflexus aurantiacus. J Bacteriol 159: 222-227.

45. Frigaard NU, Maresca JA, Yunker CE, Jones AD, Bryant DA (2004) Genetic manipulation of carotenoid biosynthesis in the green sulfur bacterium Chlorobium tepidum. J Bacteriol 186: 5210-5220.

46. Bazylinski DA, Palome E, Blakemore NA, Blakemore RP (1986) Denitrification by Chromobacterium violaceum. Appl Environ Microbiol 52: 696-699.

47. Holt JG (1986) Bergey's Manual of Systematic Bacteriology: Gram-positive Bacteria other than Actinomycetes

2. Baltimore: Williams & WilkinsHolt, J.G.

48. Bryant AE, Stevens DL (1997) The pathogenesis of gas gangrene. The clostridia: molecular biology and pathogenesis. San Diego: Academic Press. pp. 185-196.

49. Graham AF, Mason DR, Peck MW (1996) Predictive model of the effect of temperature, pH and sodium chloride on growth from spores of non-proteolytic Clostridium botulinum. International Journal of Food Microbiology 31: 69-85.

50. Heinzen RA, Hackstadt T, Samuel JE (1999) Developmental biology of Coxiella burnettii. Trends Microbiol 7: 149-154.

51. Cocaign-Bousquet M, Guyonvarch A, Lindley ND, Guyonvarch A, Lindley ND (1996) Growth Rate-Dependent Modulation of Carbon Flux through Central Metabolism and the Kinetic Consequences for Glucose-Limited Chemostat Cultures of Corynebacterium glutamicum. Appl Environ Microbiol 62: 429-436.

52. Nogi Y, Hosoya S, Kato C, Horikoshi K (2004) Colwellia piezophila sp. nov., a novel piezophilic species from deep-sea sediments of the Japan Trench. Int J Syst Evol Microbiol 54: 1627-1631.

53. Muller RH, Kleinsteuber S, Babel W (2001) Physiological and genetic characteristics of two bacterial strains utilizing phenoxypropionate and phenoxyacetate herbicides. Microbiol Res 156: 121-131.

54. Maymo-Gatell X, Chien Y, Gossett JM, Zinder SH (1997) Isolation of a bacterium that reductively dechlorinates tetrachloroethene to ethene. Science 276: 1568-1571.

55. Knoblauch C, Sahm K, Jorgensen BB (1999) Psychrophilic sulfate-reducing bacteria isolated from permanently cold Arctic marine sediments: description of Desulfofrigrus oceanense gen. nov., sp nov., Desulfofrigus fragile sp nov., Desulfofaba gelida gen. nov., sp nov., Desulfotalea psychrophila gen. nov., sp nov and Desulfotalea arctica sp nov. International Journal of Systematic Bacteriology 49: 1631-1643.

56. Harris DR, Tanaka M, Saveliev SV, Jolivet E, Earl AM, et al. (2004) Preserving genome integrity: the DdrA protein of Deinococcus radiodurans R1. PLoS Biol 2: e304.

57. Tebo BM, Obraztsova AY (1998) Sulfate-reducing bacterium grows with Cr(VI), U(VI), Mn(IV), and Fe(III) as electron acceptors. Fems Microbiology Letters 162: 193-198.

58. Pohorelic BK, Voordouw JK, Lojou E, Dolla A, Harder J, et al. (2002) Effects of deletion of genes encoding Fe-only hydrogenase of Desulfovibrio vulgaris Hildenborough on hydrogen and lactate metabolism. J Bacteriol 184: 679-686.

59. Biebl H, Allgaier M, Tindall BJ, Koblizek M, Lunsdorf H, et al. (2005) Dinoroseobacter shibae gen. nov., sp nov., a new aerobic phototrophic bacterium isolated from dinoflagellates. International Journal of Systematic and Evolutionary Microbiology 55: 1089-1096.

60. Branger S, Rolain JM, Raoult D (2004) Evaluation of antibiotic susceptibilities of Ehrlichia canis, Ehrlichia chaffeensis, and Anaplasma phagocytophilum by real-time PCR. Antimicrob Agents Chemother 48: 4822-4828.

61. Sarantinopoulos P, Makras L, Vaningelgem F, Kalantzopoulos G, De Vuyst L, et al. (2003) Growth and energy generation by Enterococcus faecium FAIR-E 198 during citrate metabolism. Int J Food Microbiol 84: 197-206.

62. Iversen C, Lane M, Forsythe SJ (2004) The growth profile, thermotolerance and biofilm formation of <i>Enterobacter sakazakii</i> grown in infant formula milk. Letters in Applied Microbiology 38: 378-382.

63. Ding F, Noritomi H, Nagahama K (2001) Optimization of Fermentation Conditions for Preparation of Polygalacturonic Acid Transeliminase by Erwinia carotovora IFO3830. Biotechnol Prog 17: 311-317.

64. Rubin LG (1986) Comparison of in vivo and in vitro multiplication rates of Haemophilus influenzae type b. Infect Immun 52: 911-913.

65. Hall JD, Craven RR, Fuller JR, Pickles RJ, Kawula TH (2007) Francisella tularensis replicates within alveolar type II epithelial cells in vitro and in vivo following inhalation. Infect Immun 75: 1034-1039.

66. Cord-Ruwisch R, Lovley DR, Schink B (1998) Growth of geobacter sulfurreducens with acetate in syntrophic cooperation with hydrogen-oxidizing anaerobic partners. Appl Environ Microbiol 64: 2232-2236.

67. Bonomi A, Fleury AT, Augusto EFP, Mattos MN, Magossi LR (1997) Mathematical modeling, automation and control of the bioconversion of sorbitol to sorbose in the vitamic C production process. I. Mathematical modeling. Braz J Chem Eng 14: ISSN 0104-6632.

68. Ussery DW (2004) Genome Update: 161 prokaryotic genomes sequenced, and counting. Microbiology 150: 261-263.

69. Bauer M, Kube M, Teeling H, Richter M, Lombardot T, et al. (2006) Whole genome analysis of the marine Bacteroidetes 'Gramella forsetii' reveals adaptations to degradation of polymeric organic matter. Environmental Microbiology 8: 2201-2213.

70. Trees DL, Morse SA (1995) Chancroid and Haemophilus ducreyi: an update. Clin Microbiol Rev 8: 357-375.

71. Johnsen U, Schonheit P (2004) Novel xylose dehydrogenase in the halophilic archaeon Haloarcula marismortui. J Bacteriol 186: 6198-6207.

72. Woodson JD, Peck RF, Krebs MP, Escalante-Semerena JC (2003) The cobY gene of the archaeon Halobacterium sp. strain NRC-1 is required for de novo cobamide synthesis. J Bacteriol 185: 311-316.

73. Bolhuis H, Poele EMT, Rodriguez-Valera F (2004) Isolation and cultivation of Walsby's square archaeon. Environmental Microbiology 6: 1287-1291.

74. Trick I, Lingens F (1984) Characterization of Herpetosiphon-Spec - a Gliding Filamentous Bacterium from Bulking-Sludge. Applied Microbiology and Biotechnology 19: 191-198.

75. Mehta NS, Benoit S, Mysore JV, Sousa RS, Maier RJ (2005) Helicobacter hepaticus hydrogenase mutants are deficient in hydrogen-supported amino acid uptake and in causing liver lesions in A/J mice. Infect Immun 73: 5311-5318.

76. Vega AE, Cortinas TI, Mattana CM, Silva HJ, Puig De Centorbi O (2003) Growth of Helicobacter pylori in medium supplemented with cyanobacterial extract. J Clin Microbiol 41: 5384-5388.

77. Jahn U, Gallenberger M, Paper W, Junglas B, Eisenreich W, et al. (2008) Nanoarchaeum equitans and Ignicoccus hospitalis: new insights into a unique, intimate association of two archaea. J Bacteriol 190: 1743-1750.

78. Adamberg K, Kask S, Laht TM, Paalme T (2003) The effect of temperature and pH on the growth of lactic acid bacteria: a pH-auxostat study. Int J Food Microbiol 85: 171-183.

79. van der Kaaij H, Desiere F, Mollet B, Germond JE (2004) L-alanine auxotrophy of Lactobacillus johnsonii as demonstrated by physiological, genomic, and gene complementation approaches. Appl Environ Microbiol 70: 1869-1873.

80. Andersen HW, Solem C, Hammer K, Jensen PR (2001) Twofold reduction of phosphofructokinase activity in Lactococcus lactis results in strong decreases in growth rate and in glycolytic flux. J Bacteriol 183: 3458-3467.

81. Barcena JM, Sineriz F, Gonzalez de Llano D, Rodriguez A, Suarez JE (1998) Chemostat production of plantaricin C by Lactobacillus plantarum LL441. Appl Environ Microbiol 64: 3512-3514.

82. Shenberg E (1967) Growth of pathogenic Leptospira in chemically defined media. J Bacteriol 93: 1598-1606.

83. Saito A, Rolfe RD, Edelstein PH, Finegold SM (1981) Comparison of liquid growth media for Legionella pneumophila. J Clin Microbiol 14: 623-627.

84. Turner JT, Lampel JS, Stearman RS, Sundin GW, Gunyuzlu P, et al. (1991) Stability of the delta-endotoxin gene from Bacillus thuringiensis subsp. kurstaki in a recombinant strain of Clavibacter xyli subsp. cynodontis. Appl Environ Microbiol 57: 3522-3528.

85. Houtsma PC, Kant-Muermans ML, Rombouts FM, Zwietering MH (1996) Model for the combined effects of temperature, pH, and sodium lactate on growth rates of Listeria innocua in broth and Bologna-type sausages. Appl Environ Microbiol 62: 1616-1622.

86. Song H, Lee JW, Choi S, You JK, Hong WH, et al. (2007) Effects of dissolved CO2 levels on the growth of Mannheimia succiniciproducens and succinic acid production. Biotechnol Bioeng 98: 1296-1304.

87. Mazumder TK, Nishio N, Fukuzaki S, Nagai S (1986) Effect of Sulfur-Containing Compounds on Growth of Methanosarcina barkeri in Defined Medium. Appl Environ Microbiol 52: 617-622.

88. Goodchild A, Saunders NF, Ertan H, Raftery M, Guilhaus M, et al. (2004) A proteomic determination of cold adaptation in the Antarctic archaeon, Methanococcoides burtonii. Mol Microbiol 53: 309-321.

89. Joergensen L, Degn H (1987) Growth-Rate and Methane Affinity of a Turbidostatic and Oxystatic Continuous Culture of Methylococcus-Capsulatus (Bath). Biotechnology Letters 9: 71-76.

90. Van Dien SJ, Okubo Y, Hough MT, Korotkova N, Taitano T, et al. (2003) Reconstruction of C(3) and C(4) metabolism in Methylobacterium extorquens AM1 using transposon mutagenesis. Microbiology 149: 601-609.

91. Brenner DJ, Staley JT (2005) Methylobacillus. Bergey’s Manual of Systematic Bacteriology. New York: Springer. pp. 771-773.

92. Rospert S, Breitung J, Ma K, Schwörer B, Zirngibl C, et al. (1991) Methyl-coenzyme M reductase and other enzymes involved in methanogenesis from CO2 and H2 in the extreme thermophile Methanopyrus kandleri. Archives of Microbiology 156: 49-55.

93. Fulchieri MM, Estrella MJ, Iglesias AA (2001) Characterization of Rhizobium loti strains from the Salado River Basin. Antonie Van Leeuwenhoek 79: 119-125.

94. Schönheit P, Moll J, Thauer RK (1980) Growth parameters (Ks, μmax, Ys) of Methanobacterium thermoautotrophicum. Archives of Microbiology 127: 59-65.

95. Byrer DE, Rainey FA, Wiegel J (2000) Novel strains of Moorella thermoacetica form unusually heat-resistant spores. Archives of Microbiology 174: 334-339.

96. Falkinham JO, 3rd (2003) Factors influencing the chlorine susceptibility of Mycobacterium avium, Mycobacterium intracellulare, and Mycobacterium scrofulaceum. Appl Environ Microbiol 69: 5685-5689.

97. Dunn PL, North RJ (1995) Virulence Ranking of Some Mycobacterium-Tuberculosis and Mycobacterium-Bovis Strains According to Their Ability to Multiply in the Lungs, Induce Lung Pathology, and Cause Mortality in Mice. Infection and Immunity 63: 3428-3437.

98. Maniloff J (1992) Mycoplasmas : molecular biology and pathogenesis / editor in chief Jack Maniloff. Washington : American Society for Microbiology.

99. Peterson SN, Fraser CM (2001) The complexity of simplicity. Genome Biol 2: COMMENT2002.

100. Madsen ML, Nettleton D, Thacker EL, Minion FC (2006) Transcriptional profiling of Mycoplasma hyopneumoniae during iron depletion using microarrays. Microbiology 152: 937-944.

101. Cole ST, Eiglmeier K, Parkhill J, James KD, Thomson NR, et al. (2001) Massive gene decay in the leprosy bacillus. Nature 409: 1007-1011.

102. Jaffe JD, Stange-Thomann N, Smith C, DeCaprio D, Fisher S, et al. (2004) The complete genome and proteome of Mycoplasma mobile. Genome Research 14: 1447-1461.

103. Teachman AM, French CT, Yu HL, Simmons WL, Dybvig K (2002) Gene transfer in Mycoplasma pulmonis. Journal of Bacteriology 184: 947-951.

104. Julien B, Shah S (2002) Heterologous expression of epothilone biosynthetic genes in Myxococcus xanthus. Antimicrob Agents Chemother 46: 2772-2778.

105. Huber H, Hohn MJ, Stetter KO, Rachel R (2003) The phylum Nanoarchaeota: Present knowledge and future perspectives of a unique form of life. Research in Microbiology 154: 165-171.

106. Hopper S, Vasquez B, Merz A, Clary S, Wilbur JS, et al. (2000) Effects of the immunoglobulin A1 protease on Neisseria gonorrhoeae trafficking across polarized T84 epithelial monolayers. Infection and Immunity 68: 906-911.

107. Deeudom M, Koomey M, Moir JWB (2008) Roles of c-type cytochromes in respiration in Neisseria meningitidis. Microbiology-Sgm 154: 2857-2864.

108. Brenner DJ, Staley JT (2005) Nitrobacter. Bergey’s Manual of Systematic Bacteriology. New York: Springer. pp. 461-467.

109. Heinzen RJ, Ensign JC (1975) Effect of growth substrates on morphology of Nocardia corallina. Arch Microbiol 103: 209-217.

110. Haselkorn R, Buikema WJ (1992) Nitrogen fixation in Cyanobacteria. In: Stacey GS, Burris RH, Evans HJ, editors. Biological Nitrogen Fixation: Springer. pp. 166-190.

111. Zhang DS, Lovitt RW (2005) Studies on growth and metabolism of Oenococcus oeni on sugars and sugar mixtures. Journal of Applied Microbiology 99: 565-572.

112. Blaszczyk M (1993) Effect of Medium Composition on the Denitrification of Nitrate by Paracoccus denitrificans. Appl Environ Microbiol 59: 3951-3953.

113. Xia X, Wei T, Xie Z, Danchin A (2002) Genomic changes in nucleotide and dinucleotide frequencies in Pasteurella multocida cultured under high temperature. Genetics 161: 1385-1394.

114. Greub G, Mege JL, Raoult D (2003) Parachlamydia acanthamoebae enters and multiplies within human macrophages and induces their apoptosis [corrected]. Infect Immun 71: 5979-5985.

115. Lien T, Madsen M, Rainey FA, Birkeland NK (1998) Petrotoga mobilis sp. nov., from a North Sea oil-production well. International Journal of Systematic Bacteriology 48: 1007-1013.

116. Bidle KA, Bartlett DH (1999) RecD function is required for high-pressure growth of a deep-sea bacterium. J Bacteriol 181: 2330-2337.

117. Rabus R, Gade D, Helbig R, Bauer M, Glockner FO, et al. (2002) Analysis of N-acetylglucosamine metabolism in the marine bacterium Pirellula sp. strain 1 by a proteomic approach. Proteomics 2: 649-655.

118. Schleper C, Puehler G, Holz I, Gambacorta A, Janekovic D, et al. (1995) Picrophilus Gen-Nov, Fam-Nov - a Novel Aerobic, Heterotrophic, Thermoacidophilic Genus and Family Comprising Archaea Capable of Growth around Ph-0. Journal of Bacteriology 177: 7050-7059.

119. Marsh PD, McDermid AS, McKee AS, Baskerville A (1994) The effect of growth rate and haemin on the virulence and proteolytic activity of Porphyromonas gingivalis W50. Microbiology 140: 861-865.

120. Kunishima S, Inoue C, Kamiya T, Ozawa K (2001) Presence of Propionibacterium acnes in blood components. Transfusion 41: 1126-1129.

121. Shalapyonok A, Olson RJ, Shalapyonok LS (1998) Ultradian Growth in Prochlorococcus spp. Appl Environ Microbiol 64: 1066-1069.

122. Medigue C, Krin E, Pascal G, Barbe V, Bernsel A, et al. (2005) Coping with cold: the genome of the versatile marine Antarctica bacterium Pseudoalteromonas haloplanktis TAC125. Genome Res 15: 1325-1335.

123. Breezee J, Cady N, Staley JT (2004) Subfreezing growth of the sea ice bacterium "Psychromonas ingrahamii". Microb Ecol 47: 300-304.

124. Ahn IS, Ghiorse WC, Lion LW, Shuler ML (1998) Growth kinetics of Pseudomonas putida G7 on naphthalene and occurrence of naphthalene toxicity during nutrient deprivation. Biotechnol Bioeng 59: 587-594.

125. Keith LM, Partridge JE, Bender CL (1999) dnaK and the heat stress response of Pseudomonas syringae pv. glycinea. Mol Plant Microbe Interact 12: 563-574.

126. Niggemyer A, Spring S, Stackebrandt E, Rosenzweig RF (2001) Isolation and characterization of a novel As(V)-reducing bacterium: implications for arsenic mobilization and the genus Desulfitobacterium. Appl Environ Microbiol 67: 5568-5580.

127. Marangoni C, Furigo J, Aragao GMF (2001) The influence of substrate source on the growth of Ralstonia eutropha, aiming at the production of polyhydroxyalkanoate. Braz J Chem Eng 18: 175-180.

128. Ramana CV, Sasikala C, Arunasri K, Kumar PA, Srinivas TNR, et al. (2006) Rubrivivax benzoatilyticus sp nov., an aromatic, hydrocarbon-degrading purple betaproteobacterium. International Journal of Systematic and Evolutionary Microbiology 56: 2157-2164.

129. Oda Y, Meijer WG, Gibson JL, Gottschal JC, Forney LJ (2004) Analysis of diversity among 3-chlorobenzoate-degrading strains of Rhodopseudomonas palustris. Microb Ecol 47: 68-79.

130. Navarro-Llorens JM, Patrauchan MA, Stewart GR, Davies JE, Eltis LD, et al. (2005) Phenylacetate catabolism in Rhodococcus sp. strain RHA1: a central pathway for degradation of aromatic compounds. J Bacteriol 187: 4497-4504.

131. Kerby RL, Ludden PW, Roberts GP (1995) Carbon Monoxide-Dependent Growth of Rhodospirillum-Rubrum. Journal of Bacteriology 177: 2241-2244.

132. Addlesee HA, Hunter CN (1999) Physical mapping and functional assignment of the geranylgeranyl-bacteriochlorophyll reductase gene, bchP, of Rhodobacter sphaeroides. J Bacteriol 181: 7248-7255.

133. Oaks SC, Jr., Osterman JV (1979) The influence of temperature and pH on the growth of Rickettsia conorii in irradiated mammalian cells. Acta Virol 23: 67-72.

134. Pang H, Winkler HH (1994) The concentrations of stable RNA and ribosomes in Rickettsia prowazekii. Mol Microbiol 12: 115-120.

135. Higgins JA, Radulovic S, Noden BH, Troyer JM, Azad AF (1998) Reverse transcriptase PCR amplification of Rickettsia typhi from infected mammalian cells and insect vectors. J Clin Microbiol 36: 1793-1794.

136. Empadinhas N, Mendes V, Simões C, Santos M, Mingote A, et al. (2007) Organic solutes in Rubrobacter xylanophilus : the first example of di- myo -inositol-phosphate in a thermophile. Extremophiles 11: 667-673.

137. Anton J, Oren A, Benlloch S, Rodriguez-Valera F, Amann R, et al. (2002) Salinibacter ruber gen. nov., sp nov., a novel, extremely halophilic member of the Bacteria from saltern crystallizer ponds. International Journal of Systematic and Evolutionary Microbiology 52: 485-491.

138. Abboud R, Popa R, Souza-Egipsy V, Giometti CS, Tollaksen S, et al. (2005) Low-temperature growth of Shewanella oneidensis MR-1. Appl Environ Microbiol 71: 811-816.

139. Ampe F, Kiss E, Sabourdy F, Batut J (2003) Transcriptome analysis of Sinorhizobium meliloti during symbiosis. Genome Biol 4: R15.

140. Gonzalez JM, Covert JS, Whitman WB, Henriksen JR, Mayer F, et al. (2003) Silicibacter pomeroyi sp. nov. and Roseovarius nubinhibens sp. nov., dimethylsulfoniopropionate-demethylating bacteria from marine environments. Int J Syst Evol Microbiol 53: 1261-1269.

141. Matthew CZ, Darby AC, Young SA, Hume LH, Welburn SC (2005) The rapid isolation and growth dynamics of the tsetse symbiont Sodalis glossinidius. FEMS Microbiol Lett 248: 69-74.

142. Fegatella F, Lim J, Kjelleberg S, Cavicchioli R (1998) Implications of rRNA operon copy number and ribosome content in the marine oligotrophic ultramicrobacterium Sphingomonas sp. strain RB2256. Applied and Environmental Microbiology 64: 4433-4438.

143. Malin G, Paoletti LC (2001) Use of a dynamic in vitro attachment and invasion system (DIVAS) to determine influence of growth rate on invasion of respiratory epithelial cells by group B Streptococcus. Proc Natl Acad Sci U S A 98: 13335-13340.

144. Somerville GA, Said-Salim B, Wickman JM, Raffel SJ, Kreiswirth BN, et al. (2003) Correlation of acetate catabolism and growth yield in Staphylococcus aureus: implications for host-pathogen interactions. Infect Immun 71: 4724-4732.

145. Shahab N, Flett F, Oliver SG, Butler PR (1996) Growth rate control of protein and nucleic acid content in Streptomyces coelicolor A3(2) and Escherichia coli B/r. Microbiology 142: 1927-1935.

146. Gottenbos B, van der Mei H, Busscher H (2000) Initial adhesion and surface growth of Staphylococcus epidermidis and Pseudomonas aeruginosa on biomedical polymers. J Biomed Materials Res 50: 208-214.

147. Hao X, Ma K (2003) Minimal sulfur requirement for growth and sulfur-dependent metabolism of the hyperthermophilic archaeon Staphylothermus marinus. Archaea 1: 191-197.

148. Shimamoto T, Fukui K, Kodama T, Shimono T, Ohta H, et al. (1990) Effects of oxygen on growth of Streptococcus mutans. Shika Kiso Igakkai Zasshi 32: 10-19.

149. Small PM, Tauber MG, Hackbarth CJ, Sande MA (1986) Influence of body temperature on bacterial growth rates in experimental pneumococcal meningitis in rabbits. Infect Immun 52: 484-487.

150. Biswas I, Germon P, McDade K, Scott JR (2001) Generation and surface localization of intact M protein in Streptococcus pyogenes are dependent on sagA. Infect Immun 69: 7029-7038.

151. O'Sullivan TF, Fitzgerald GF (1999) Electrotransformation of industrial strains of Streptococcus thermophilus. J Appl Microbiol 86: 275-283.

152. McInerney MJ, Rohlin L, Mouttaki H, Kim U, Krupp RS, et al. (2007) The genome of Syntrophus aciditrophicus: life at the thermodynamic limit of microbial growth. Proc Natl Acad Sci U S A 104: 7600-7605.

153. Katayama M, Kondo T, Xiong J, Golden SS (2003) ldpA encodes an iron-sulfur protein involved in light-dependent modulation of the circadian period in the cyanobacterium Synechococcus elongatus PCC 7942. J Bacteriol 185: 1415-1422.

154. Harmsen HJM, Van Kuijk BLM, Plugge CM, Akkermans ADL, De Vos WM, et al. (1998) Syntrophobacter fumaroxidans sp. nov., a syntrophic propionate-degrading sulfate-reducing bacterium. International Journal of Systematic Bacteriology 48: 1383-1387.

155. Johnson TW, Shen G, Zybailov B, Kolling D, Reategui R, et al. (2000) Recruitment of a foreign quinone into the A(1) site of photosystem I. I. Genetic and physiological characterization of phylloquinone biosynthetic pathway mutants in Synechocystis sp. pcc 6803. J Biol Chem 275: 8523-8530.

156. Ohno M, Okano I, Watsuji T, Kakinuma T, Ueda K, et al. (1999) Establishing the independent culture of a strictly symbiotic bacterium Symbiobacterium thermophilum from its supporting Bacillus strain. Biosci Biotechnol Biochem 63: 1083-1090.

157. Mori T, Binder B, Johnson CH (1996) Circadian gating of cell division in cyanobacteria growing with average doubling times of less than 24 hours. Proc Natl Acad Sci U S A 93: 10183-10188.

158. Jannasch HW, Wirsen CO, Nelson DC, Robertson LA (1985) Thiomicrospira-Crunogena Sp-Nov, a Colorless, Sulfur-Oxidizing Bacterium from a Deep-Sea Hydrothermal Vent. International Journal of Systematic Bacteriology 35: 422-424.

159. Iwai M, Katayama M, Ikeuchi M (2006) Absence of the psbH gene product destabilizes the Photosystem II complex and prevents association of the Photosystem II-X protein in the thermophilic cyanobacterium Thermosynechococcus elongatus BP-1. Photosynthesis Research 87: 313-322.

160. Chhabra SR, Shockley KR, Ward DE, Kelly RM (2002) Regulation of endo-acting glycosyl hydrolases in the hyperthermophilic bacterium Thermotoga maritima grown on glucan- and mannan-based polysaccharides. Appl Environ Microbiol 68: 545-554.

161. Xue Y, Xu Y, Liu Y, Ma Y, Zhou P (2001) Thermoanaerobacter tengcongensis sp. nov., a novel anaerobic, saccharolytic, thermophilic bacterium isolated from a hot spring in Tengcong, China. Int J Syst Evol Microbiol 51: 1335-1341.

162. Demirtas MU, Kolhatkar A, Kilbane JJ, 2nd (2003) Effect of aeration and agitation on growth rate of Thermus thermophilus in batch mode. J Biosci Bioeng 95: 113-117.

163. Dollhopf SL, Pariseau ML, Hashsham SA, Tiedje JM (2003) Competitive and interactions affecting a fermentative spirochete in anaerobic chemostats. Microb Ecol 46: 1-11.

164. El-Shehawy R, Lugomela C, Ernst A, Bergman B (2003) Diurnal expression of hetR and diazocyte development in the filamentous non-heterocystous cyanobacterium Trichodesmium erythraeum. Microbiology 149: 1139-1146.

165. Cox DL, Riley B, Chang P, Sayahtaheri S, Tassell S, et al. (1990) Effects of molecular oxygen, oxidation-reduction potential, and antioxidants upon in vitro replication of Treponema pallidum subsp. pallidum. Appl Environ Microbiol 56: 3063-3072.

166. Renesto P, Crapoulet N, Ogata H, La Scola B, Vestris G, et al. (2003) Genome-based design of a cell-free culture medium for Tropheryma whipplei. Lancet 362: 447-449.

167. McFall-Ngai MJ (2000) Negotiations between animals and bacteria: the 'diplomacy' of the squid-vibrio symbiosis. Comp Biochem Physiol A Mol Integr Physiol 126: 471-480.

168. Twedt RM, Novelli RM (1971) Modified selective and differential isolation medium for Vibrio parahaemolyticus. Appl Microbiol 22: 593-599.

169. Fenollar F, Maurin M, Raoult D (2003) Wolbachia pipientis growth kinetics and susceptibilities to 13 antibiotics determined by immunofluorescence staining and real-time PCR. Antimicrob Agents Chemother 47: 1665-1671.

170. Ullmann R, Gross R, Simon J, Unden G, Kroger A (2000) Transport of C(4)-dicarboxylates in Wolinella succinogenes. J Bacteriol 182: 5757-5764.

171. Wichmann G, Ritchie D, Kousik CS, Bergelson J (2005) Reduced genetic variation occurs among genes of the highly clonal plant pathogen Xanthomonas axonopodis pv. vesicatoria, including the effector gene avrBs2. Appl Environ Microbiol 71: 2418-2432.

172. Pena C, Galindo E, Diaz M (2002) Effectiveness factor in biological external convection: study in high viscosity systems. J Biotechnol 95: 1-12.

173. Ehrlich M, Lin FH, Ehrlich K, Brown SL, Mayo JA (1977) Changes in macromolecular synthesis in Xanthomonas oryzae infected with bacteriophage XP-12. J Virol 23: 517-523.

174. Feil H, Purcell AH (2001) Temperature-dependent growth and survival of Xylella fastidiosa in vitro and in potted grapevines. Plant Disease 85: 1230-1234.

175. Chu M (2001) Basic laboratory protocols for the presumptive identification of Yersinia pestis

Tertiary Basic laboratory protocols for the presumptive identification of Yersinia pestis. Atlanta. 1-19.

176. Dreyfus LA, Brubaker RR (1978) Consequences of aspartase deficiency in Yersinia pestis. J Bacteriol 136: 757-764.

177. Sprenger GA, Typas MA, Drainas C (1993) Genetics and Genetic-Engineering of Zymomonas-Mobilis. World Journal of Microbiology & Biotechnology 9: 17-24.
