# Supplementary material for: The Systemic Imprint of Growth and Its Uses in Ecological (Meta)Genomics
Source: PLoS Genet. 2010 Jan 15;6(1):e1000808. doi: 10.1371/journal.pgen.1000808 (PMC2797632; doi:10.1371/journal.pgen.1000808)
Supplement: Table S2 — List of ubiquitous tRNAs (ubi-tRNA) in 102 bacterial species, previously published [35]. (0.04 MB DOC) [file pgen.1000808.s006.doc]

**Supplementary Table 2**: **List of ubiquitous tRNAs (ubi-tRNA) in 102 bacterial species, previously published** [35].

| **Amino acid** | **Number of codons** | **Most frequent anticodon** |
| --- | --- | --- |
| Met | 1 | CAU |
| Trp | 1 | CCA |
| Ile | 3 | GAU |
| Asn | 2 | GUU |
| Asp | 2 | GUC |
| Cys | 2 | GCA |
| His | 2 | GUG |
| Phe | 2 | GAA |
| Tyr | 2 | GUA |
| Glu | 2 | UUC |
| Gln | 2 | UUG |
| Lys | 2 | UUU |
| Ala | 4 | UGC |
| Gly | 4 | GCC |
| Val | 4 | UAC |
| Pro | 4 | UGG |
| Thr | 4 | UGU |
| Arg_2_ | 2 | UCU |
| Arg_4_ | 4 | ACG |
| Ser_2_ | 2 | GCU |
| Ser_4_ | 4 | UGA |
| Leu_2_ | 2 | UAA |
| Leu_4_ | 4 | UAG |
