# Supplementary material for: The Systemic Imprint of Growth and Its Uses in Ecological (Meta)Genomics
Source: PLoS Genet. 2010 Jan 15;6(1):e1000808. doi: 10.1371/journal.pgen.1000808 (PMC2797632; doi:10.1371/journal.pgen.1000808)
Supplement: Table S3 — Most informative attributes for minimum generation time prediction. The results of a stepwise forward regression are given, where the most informative attributes enter first. Individual and cumulative coefficients of determination (R2) are given for the 10 genomic attributes under study and one extra attribute: the minimum generation time of the closest organism in our 16S phylogenetic tree. Individual and cumulative R2 are, respectively, the fraction of the variance of d explained by the variable alone and by the variable combined with all the variables above in the table (N = 188). The p-values before and after phylogenetic dependency correction are given for the individual R2. Species with unknown origins of replication were excluded. (0.04 MB DOC) [file pgen.1000808.s007.doc]

**Supplementary Table 3: Most informative attributes for minimum generation time prediction.** The results of a stepwise forward regression are given, where the most informative attributes enter first. Individual and cumulative coefficients of determination (R^2^) are given for the 10 genomic attributes under study and one extra attribute: the minimum generation time of the closest organism in our 16S phylogenetic tree. Individual and cumulative R^2^ are, respectively, the fraction of the variance of d explained by the variable alone and by the variable combined with all the variables above in the table (N=188). The p-values before and after phylogenetic dependency correction are given for the individual R^2^. Species with unknown origins of replication were excluded.

| Variable | **Individual ρ** | **Individual R^2^** | **Cumulative R^2^** | **Order** | **Ordered contribution R^2^** |
| --- | --- | --- | --- | --- | --- |
| ΔENC’ ^A^ | -0.70^++^ | 0.50^++/**^ | 0.50^++^ | 1 | 0.50^++^ |
| Closest species d ^D^ | 0.68^++^ | 0.47^++^ | 0.60^++^ | 2 | 0.10^++^ |
| S ^A^ | -0.60^++^ | 0.39^++/**^ | 0.63^++^ | 3 | 0.03^++^ |
| rRNA position ^B^ | 0.36^++^ | 0.15^++/**^ | 0.64^+^ | 4 | 0.01^+^ |
| ubi-tRNA position ^B^ | 0.41^++^ | 0.21^++/NS^ | 0.65 | 5 | NS |
| rRNA number ^C^ | -0.66^++^ | 0.41^++/**^ | 0.65 | 6 | NS |
| tRNA position ^B^ | 0.35^++^ | 0.18^++/*^ | 0.65 | 7 | NS |
| tRNA number ^C^ | -0.59^++^ | 0.33^++/**^ | 0.65 | 8 | NS |
| ubi-tRNA number ^C^ | -0.68^++^ | 0.40^++/**^ | 0.65 | 9 | NS |
| rpol position ^B^ | 0.42^++^ | 0.18^++/**^ | 0.65 | 10 | NS |
| rp position ^B^ | 0.42^++^ | 0.17^++/**^ | 0.65 | 11 | NS |

^A^ codon usage bias effects; ^B^ replication-associated gene dosage effects; ^C^ gene multiplicity effects; ^D^ closest organism minimum generation time.

NS: non-significant p-value; ^++^ p-value<0.001; ^+^ p-value<0.05

After phylogenetic dependency correction: ^**^ p-value<0.001; ^*^ p-value<0.05; NS: non-significant p-value
