# Supplementary material for: The Systemic Imprint of Growth and Its Uses in Ecological (Meta)Genomics
Source: PLoS Genet. 2010 Jan 15;6(1):e1000808. doi: 10.1371/journal.pgen.1000808 (PMC2797632; doi:10.1371/journal.pgen.1000808)
Supplement: Table S4 — Accuracy of a discrete classification of the 187 mesophilic species. Classification into 4 classes: very fast (d<1h, N = 46), fast (1h<d<2h, N = 26), intermediate (2h<d<5h, N = 41) and slow (d≥5h, N = 74). Proportion of exact, approximate and wrong classifications (%), respectively defined as the proportion of 1) predictions matching the same observed class, 2) predictions matching the same observed class or the adjacent ones (e.g. predicted ‘fast’ when actually ‘very fast’) and 3) slow growers predicted as fast or very fast and inversely. Genes were chosen randomly in the complete subsets (ribosomal proteins (HEG) or other proteins (non-HEG)) for 1000 random experiments. (0.03 MB DOC) [file pgen.1000808.s008.doc]

**Supplementary Table 4**: **Accuracy of a discrete classification of the 187 mesophilic species**. Classification into 4 classes: very fast (d<1h, N=46), fast (1h<d<2h, N=26), intermediate (2h<d<5h, N=41) and slow (d≥5h, N=74). Proportion of exact, approximate and wrong classifications (%), respectively defined as the proportion of 1) predictions matching the same observed class, 2) predictions matching the same observed class or the adjacent ones (e.g. predicted ‘fast’ when actually ‘very fast’) and 3) slow growers predicted as fast or very fast and inversely. Genes were chosen randomly in the complete subsets (ribosomal proteins (HEG) or other proteins (non-HEG)) for 1000 random experiments.

| Number of genes used for the prediction | Classification | | |
| --- | --- | --- | --- |
|  | Exact | Approximate or exact | Wrong |
| all HEG + all non-HEG | 59% | 93% | 4% |
| 20 HEG + 20 non-HEG | 55% | 92% | 5% |
| 10 HEG + 10 non-HEG | 54% | 91% | 5% |
| 5 HEG + 5 non-HEG | 50% | 89% | 7% |
| Expected | 25% | 59% | 29% |
