# Supplementary material for: The Systemic Imprint of Growth and Its Uses in Ecological (Meta)Genomics
Source: PLoS Genet. 2010 Jan 15;6(1):e1000808. doi: 10.1371/journal.pgen.1000808 (PMC2797632; doi:10.1371/journal.pgen.1000808)
Supplement: Table S5 — Description of the metagenomes of the 3 environmental samples. (0.03 MB DOC) [file pgen.1000808.s009.doc]

**Supplementary Table 5**: **Description of the metagenomes of the 3 environmental samples.**

|  | **Acid Drainage** | **Farm Soil** | **Human Gut** |
| --- | --- | --- | --- |
| Number of contigs | 2455 | 139340 | 22508 |
| Maximum length of contigs (kbp) | 133.6 | 6.6 | 25.1 |
| Average length of contigs (kbp) | 4.2 | 1.0 | 1.4 |
| Number of genes^a^ | 10583 | 121433 | 35359 |
| Number of HEG^b^ | 165 | 445 | 416 |

^a^ genes retrieved using EMBOSS function getorf (>450bp)

^b^ Highly Expressed Genes retrieved by similarity with a database of ribosomal proteins of all sequenced genomes available to date (e-value<10^-5^).
