# Supplementary material for: The Systemic Imprint of Growth and Its Uses in Ecological (Meta)Genomics
Source: PLoS Genet. 2010 Jan 15;6(1):e1000808. doi: 10.1371/journal.pgen.1000808 (PMC2797632; doi:10.1371/journal.pgen.1000808)
Supplement: Table S6 — Description of the human gut metagenomes for 3 age groups. (0.03 MB DOC) [file pgen.1000808.s010.doc]

**Supplementary Table 6**: **Description of the human gut metagenomes for 3 age groups.**

|  | **Babies** | **Children** | **Adults** |
| --- | --- | --- | --- |
| Number of individuals | 4 | 2 | 7 |
| Number of contigs^*^ | 15798 | 33207 | 31557 |
| Maximum length of contigs (kbp) ^*^ | 25.8 | 17.6 | 21.7 |
| Average length of contigs (kbp) ^*^ | 1.5 | 1.3 | 1.3 |
| Number of genes^a*^ | 19896 | 38602 | 36046 |
| Number of HEG^b*^ | 136 | 288 | 276 |

^*^ average per individual

^a^ genes retrieved using EMBOSS function getorf (>450bp)

^b^ Highly Expressed Genes retrieved by similarity with a database of ribosomal proteins of all sequenced genomes available to date (e-value<10^-5^).
