# Supplementary material for: The Systemic Imprint of Growth and Its Uses in Ecological (Meta)Genomics
Source: PLoS Genet. 2010 Jan 15;6(1):e1000808. doi: 10.1371/journal.pgen.1000808 (PMC2797632; doi:10.1371/journal.pgen.1000808)
Supplement: Table S7 — List of the sequenced complete genomes matching the proteins of the environmental metagenomes. (0.04 MB DOC) [file pgen.1000808.s011.doc]

**Supplementary Table 7:** List of the sequenced complete genomes matching the proteins of the environmental metagenomes.

| Dataset | **Complete genome matching proteins in the dataset** |
| --- | --- |
| Acid Mine Drainage Biofilm | 1. Ferroplasma acidarmanus Type I 2. Ferroplasma sp. Type II 3. Leptospirillum sp. Group II 4. Leptospirillum sp. Group III 5. Thermoplasmatales archaeon Gp l |
| Waseca County Farm Soil | 1. Anaeromyxobacter dehalogenans 2CP-C 2. Anaeromyxobacter sp. Fw109-5 3. Arthrobacter aurescens TC1 4. Azoarcus sp. EbN1 5. Bradyrhizobium japonicum USDA 110 6. Bradyrhizobium sp. ORS278 7. Cellvibrio japonicus Ueda107 8. Escherichia coli str. K-12 substr. MG1655 9. Escherichia fergusonii ATCC 35469 10. Flavobacterium johnsoniae UW101 11. Herminiimonas arsenicoxydans 12. Methanosarcina barkeri str. fusaro 13. Mesorhizobium loti MAFF303099 14. Methanosarcina mazei Go1 15. Mycobacterium gilvum PYR-GCK 16. Nitrobacter hamburgensis X14 17. Nitrobacter winogradskyi Nb-255 18. Novosphingobium aromaticivorans DSM 12444 19. Nocardioides sp. JS614 20. Opitutus terrae PB90-1 21. Phenylobacterium zucineum HLK1 22. Polaromonas sp. JS666 23. Pseudomonas putida KT2440 24. Rhodoferax ferrireducens T118 25. Rhizobium leguminosarum bv. viciae 3841 26. Rhodopseudomonas palustris BisB18 27. Salinispora arenicola CNS-205 28. Shigella boydii Sb227 29. Shigella flexneri 2a str. 301 30. Shigella sonnei Ss046 31. Sinorhizobium meliloti 1021 32. Sorangium cellulosum 'So ce 56' 33. Variovorax paradoxus S110 |
| Human Distal Gut Metagenome | 1. Bifidobacterium adolescentis ATCC 15703 2. Bifidobacterium animalis subsp. lactis AD011 3. Bifidobacterium longum NCC2705 4. Clostridium difficile 630 5. Corynebacterium urealyticum DSM 7109 6. Enterococcus faecalis V583 7. Eubacterium eligens ATCC 27750 8. Eubacterium rectale ATCC 33656 9. Lactococcus lactis subsp. lactis Il1403 10. Methanobrevibacter smithii ATCC 35061 11. Methanosphaera stadtmanae DSM 3091 12. Lactobacillus helveticus DPC 4571 13. Streptococcus agalactiae 2603V/R 14. Streptococcus suis 05ZYH33 15. Streptococcus thermophilus LMG 18311 |
