# Supplementary material for: The Systemic Imprint of Growth and Its Uses in Ecological (Meta)Genomics
Source: PLoS Genet. 2010 Jan 15;6(1):e1000808. doi: 10.1371/journal.pgen.1000808 (PMC2797632; doi:10.1371/journal.pgen.1000808)
Supplement: Table S8 — Comparison of whole metagenome and pseudo-genome analysis for the 3 age groups human gut metagenomes. (0.03 MB DOC) [file pgen.1000808.s012.doc]

**Supplementary Table 8**: **Comparison of whole metagenome and pseudo-genome analysis for the 3 age groups human gut metagenomes.**

|  | **Predicted minimum generation time (hours)** | | | | | |
| --- | --- | --- | --- | --- | --- | --- |
|  | **Babies** | | **Children** | | Adults | |
| Whole metagenome (bootstrap) | 1.4 | A^*^ | 2.4 | B^*^ | 2.4 | B^*^ |
| Pseudo-genomes (arithmetic mean) | 1.0 | A^*^ | 1.3 | A^*^ | 1.4 | A^*^ |
| Pseudo-genomes (weighted mean)^a^ | 0.8 | A^*^ | 2.0 | B^*^ | 1.8 | B^*^ |
| Pseudo-genomes (weighted, independent)^ab^ | 1.0 | A^*^ | 2.2 | B^*^ | 1.8 | B^*^ |

^a^ The prediction for one pseudo-genome was weighted by the number of proteins from the metagenome that matched it.

^b^ To control for phylogenetic dependencies, only one representing pseudo-genome per genera was maintained (chosen randomly).

^*^ For each analysis, the groups which have significantly different predicted minimal doubling times are represented by a different letter (Tukey-Kramer: p-value<0.01).
